# Text-derived concept profiles support assessment of DNA microarray data for acute myeloid leukemia and for androgen receptor stimulation

**DOI:** 10.1186/1471-2105-8-14

**Published:** 2007-01-18

**Authors:** Rob Jelier, Guido Jenster, Lambert CJ Dorssers, Bas J Wouters, Peter JM Hendriksen, Barend Mons, Ruud Delwel, Jan A Kors

**Affiliations:** 1 Department of Medical Informatics, Erasmus MC – University Medical Center, Rotterdam, The Netherlands; 2 Department of Urology, Erasmus MC – University Medical Center, Rotterdam, The Netherlands; 3 Department of Pathology, Erasmus MC – University Medical Center, Rotterdam, The Netherlands; 4 Department of Hematology, Erasmus MC – University Medical Center, Rotterdam, The Netherlands

## Abstract

**Background:**

High-throughput experiments, such as with DNA microarrays, typically result in hundreds of genes potentially relevant to the process under study, rendering the interpretation of these experiments problematic. Here, we propose and evaluate an approach to find functional associations between large numbers of genes and other biomedical concepts from free-text literature. For each gene, a profile of related concepts is constructed that summarizes the context in which the gene is mentioned in literature. We assign a weight to each concept in the profile based on a likelihood ratio measure. Gene concept profiles can then be clustered to find related genes and other concepts.

**Results:**

The experimental validation was done in two steps. We first applied our method on a controlled test set. After this proved to be successful the datasets from two DNA microarray experiments were analyzed in the same way and the results were evaluated by domain experts. The first dataset was a gene-expression profile that characterizes the cancer cells of a group of acute myeloid leukemia patients. For this group of patients the biological background of the cancer cells is largely unknown. Using our methodology we found an association of these cells to monocytes, which agreed with other experimental evidence. The second data set consisted of differentially expressed genes following androgen receptor stimulation in a prostate cancer cell line. Based on the analysis we put forward a hypothesis about the biological processes induced in these studied cells: secretory lysosomes are involved in the production of prostatic fluid and their development and/or secretion are androgen-regulated processes.

**Conclusion:**

Our method can be used to analyze DNA microarray datasets based on information explicitly and implicitly available in the literature. We provide a publicly available tool, dubbed Anni, for this purpose.

## Background

The outcome of high-throughput experiments, such as DNA microarray experiments, is typically a list of hundreds of genes that could be relevant to the studied phenomenon. Further analysis is required to relate the genes to relevant biological processes and to identify potentially interesting relationships between the genes. In the early days of DNA microarray data analysis, extracting the required information about genes depended solely on researchers retrieving information from the huge corpus of scientific literature. Nowadays, the need for computational support in the interpretation of high-throughput experiments has become widely recognized.

However, much of the knowledge on genes and proteins is locked in unstructured free text and cannot be used directly in computational systems. To make this knowledge more accessible, several databases have become available that offer structured information on genes and proteins. These databases are either public, e.g. the databases offered by the Gene Ontology Annotation (GOA) project [1] and the Kyoto Encyclopedia of Genes and Genomes (KEGG) project [2], or corporate, e.g. as delivered by GeneGO [3] and Ingenuity [4]. For a large part, these databases are filled with manually encoded information generated by experts reading scientific literature. Manual encoding is generally considered a reliable method for extracting information from literature, but due to its labor-intensive nature it is limited in scope and flexibility. Complementary to manual encoding, research effort is currently spent on text-mining: the development of computerized algorithms for extracting information from scientific literature [5]. Automated methods have the advantage of speed and adaptability, with the challenging obligation to achieve both high precision and recall.

In text-mining, broadly two approaches can be distinguished. One approach is focused on the extraction of explicitly stated direct relationships between genes and other biomedical concepts. Early proposed systems for this task were based on the co-occurrence of terms in texts [6,7]. Currently, the grammatical structure in a sentence is typically used for the task of relation mining and a wide variety of techniques has been developed. These techniques range from the detection of simple patterns such as "protein A – action X – protein B" [8,9], to the complete parsing of whole sentences [10,11]. The other approach is focused on the identification of indirect associations between concepts, such as genes. For instance, two genes can be found to have an association, because they are described in separate papers to be involved in the same biological process. To retrieve such indirect associations, the explicit, direct associations of the genes are compared. In this approach, syntactic structures are typically ignored, and only the statistics of occurrences and co-occurrences of words or terms in a text come into play.

Here we focus on the second approach. Several co-occurrence based methods have been developed for the analysis of DNA microarray data. GEISHA [12] took a cluster of genes from a DNA microarray data analysis. The system annotated this cluster with the most discriminant terms, and also retrieved relevant co-occurrences, sentences, and abstracts. The system was word-based but automatically identified common word combinations and treated them as single concepts. Shatkay et al. [13] used a kernel document to represent a gene, and used this document to retrieve a set of similar documents. A list of keywords was generated to summarize the recurring theme in the genes' sets of retrieved documents. Subsequently, genes were associated to each other by comparing the genes' sets of retrieved documents. Raychaudhuri et al. [14] analyzed a list of genes by identifying clusters of genes that show "functional coherence" according to their literature-based neighbor divergence measure. We introduced the associative concept space (ACS) [15] as an aid to find associations between genes for microarray data analysis. The algorithm positioned concepts, in an iterative process, in a virtual space based on co-occurrence information. The idea behind the ACS is that concepts that are placed close to each other will be more likely to share an actual semantic relationship and the visualized ACS allowed browsing for associations between concepts, which is intuitively appealing. Several authors [16-20] employed the vector space model, in which a gene is represented by means of a vector that characterizes a set of texts associated with the gene. The methods varied in the features, or dimensions, of the vector. Chaussabel and Sher [17] used a simple word-based approach to generate a list of co-occurring words for each gene. For the analysis of a list of genes, they attempted to bring to light interesting co-occurrence patterns by clustering both the genes and the co-occurring words. Glennison et al. [16] used concepts from a thesaurus as features, and identified terms in texts referring to thesaurus concepts. They used five thesauri to obtain different views on the associations of a gene and used clustering to find genes with similar profiles from a gene list. Others used factorization techniques to reduce the high dimensionality encountered when using words or concepts as features: Küffner et al. and Homayouni et al. used singular value decomposition [18,19] and Chagoyen et al. employed non-negative matrix factorization [20]. The claim is that reduction of the dimensionality in this manner leads to a more robust data.-analysis, which is less sensitive to sparse and noisy data [20].

From a user's perspective, the current approaches leave several requirements unfulfilled. For example, the ACS and Raychaudhuri methods suffer from a lack of transparency, i.e., a user will not easily understand how the programs come to their associations, which is important to know in an actual research setting. Transparency is also at stake when using factorization in vector space approaches, as it is not clear what the newly defined dimensions mean, or even whether the have a semantic interpretation at all. The methods described by Glennison and Chaussabel and Sher are transparent but use empirical methods for the weighting of concepts, which have problematic statistical properties (see Discussion section for more information). Also, it would be desirable for a user to have more control on which concepts or words are used to compute an association than is possible in the mentioned approaches.

Our aim in this paper is to create a text-mining system for the interpretation of gene lists derived from DNA microarray data that is transparent. Furthermore, in contrast to many earlier published text-mining systems, we will apply the system to actual research problems, in cooperation with molecular biologists. The approach we propose finds associations between genes by means of concept (co-) occurrence statistics and employs the vector space model, similar to Glennison et al. [16]. For each gene we generate a vector of weights, which we refer to as a concept profile. The features in the concept profile are thesaurus concepts that characterize a set of documents associated with the gene. A thesaurus concept is an entity with a definition and a set of terms that are used in texts, to refer to the concept. Every concept is also assigned a semantic type, such as "disease" or "gene". The set of concepts used in the concept profiles is filtered by semantic type using a user defined semantic filter. An important issue is the selection of the measure to weigh the association of a concept in a profile. The weight should distinguish between a concept that co-occurs through chance with the concept of interest and a concept with a semantically interesting association. With this in mind we adopted a test-based method based on likelihood ratios [21], which has been successfully used for the identification of interesting collocations [22]. Compared to other test-based methods, the likelihood ratio does not require the data to have a normal distribution and is known to yield good results even on small samples. We developed a program called Anni to work with the concept profiles. With this program, genes associated with similar topics in literature are identified by hierarchical clustering of the corresponding gene concept profiles. Anni has a high degree of transparency. It provides for every identified cluster Anni a coherence measure, and also a p-value to illustrate how exceptional the cluster is, and a complete annotation of the underlying overlap of the concept profiles. Also, a link to the underlying texts is provided for all associations in the concept profiles. The program is freely available at .

We evaluated the method in two steps. Firstly, we present an evaluation based on a controlled test set and compare it to our earlier published ACS algorithm [15]. Secondly, we give a systematic analysis of the data from two DNA microarray experiments and evaluate the results together with domain experts.

## Results

### Performance evaluation on a controlled test set

**Figure 1:**
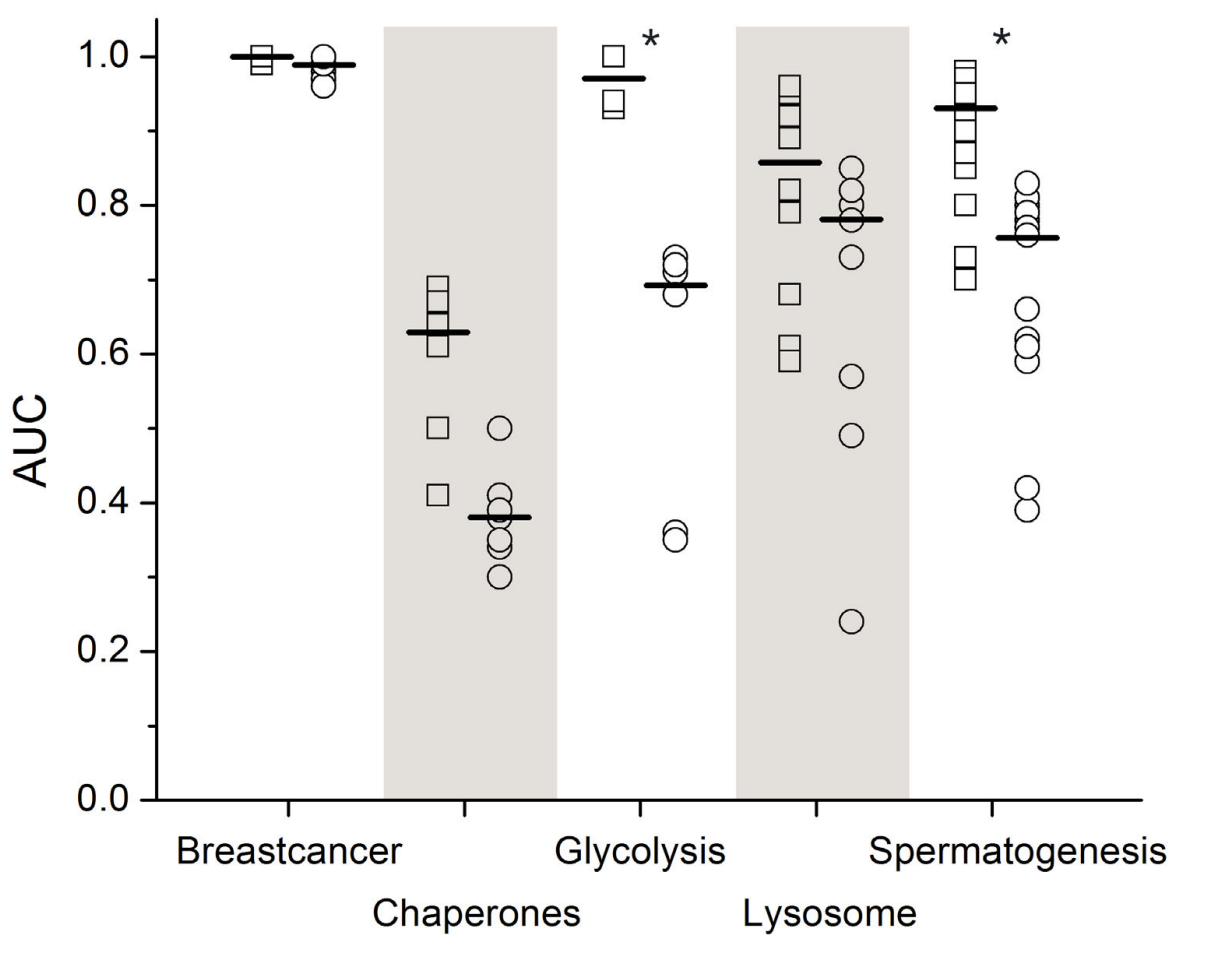
Area under the curve scores for individual genes per group for the concept profile method (open boxes) and the ACS (open circles). An asterisk above a group indicates that the difference in performance of the two methods is statistically significant (at the 0,05 level).

The concept profile method and the ACS were compared based on a controlled test set, as described before [15]. The test set was made by pooling five groups of genes that share a biological relationship: chaperone activity (7 genes), glycolysis (6), breast cancer (9), spermatogenesis (15) and lysosome (10). A table with all 47 genes is given in Additional file 1. For each gene the methods were evaluated on their ability to distinguish between group members and non-group members. Receiver operating characteristics (ROC) curves were constructed for every gene and the area under the ROC curve (AUC) supplied the evaluation measure. As can be seen in Figure 1, the concept profile method has high AUC scores for 4 out of 5 gene groups. It significantly outperforms the ACS in 2 out of 5 groups and has higher median scores for the other groups as well. Overall, taking the genes from all groups together, the concept profile method significantly outperforms the ACS (p < 0,05). As discussed in [15], the poor score for the chaperone group is caused by the scarce reference in the literature to this function. We examined with Anni the concept profiles of each gene group and looked for the ranking of the concept that characterizes the group's shared biological association. In their respective group annotation the concept "breast neoplasms" was ranked first, "lysosome" came second, "spermatogenesis" second, "molecular chaperones" first and "glycolysis" fifth. All groups, with the exception of the chaperone group, had significant cohesion scores (p < 0,05).

### DNA microarray dataset 1: Gene expression profiles of acute myeloid leukemia patients

Based on gene-expression profiles of leukemic cells, 285 acute myeloid leukemia (AML) patients were separated into 16 groups [23]. Several of these groups coincided with known classes of AML patients. AML cases are classified by the occurrence of genomic aberrations in the leukemic cells. According to the report, group 5, one of the larger groups with 61 patients, does not associate with a known karyotypic abnormality and little is known about the background of the leukemic cells in this cluster [23]. The set of genes that characterize this patient group were analyzed with the literature-based clustering provided by Anni. We sought to find shared processes and other associations that could be indicative for the background of the leukemic cells.

A total of 42 gene clusters were found for the 992 genes in patient group 5 (the complete Anni analysis is included as Additional file 2). Based on this annotation we put forward the hypothesis of an association of patient group 5 to monocytes on the following grounds: Two clusters of genes were found to be involved in phagocytosis: a cluster of cathepsins and a cluster associated with respiratory burst. Of the cathepsins, *CTSS*, *CTSB *and *CTSL *are implicated in antigen presentation on the surface of cells from the monocytic lineage [24,25]. Respiratory burst is a process characteristic for a sub-type of blood cells called phagocytes. From the group of phagocytes, we can exclude granulocytes as we identified a cluster associated with the major histocompatibility complex class 2 (MHC II). The presence of MHC II is a distinguishing factor between the myeloid cell types for it is absent in neutrophils, basophils and eosinophils [26]. This leaves us with monocytes.

Also within several other clusters genes were found to have an association with monocytes in their concept profile. Several of these genes indeed had a functional relationship with monocytes. A cluster of chemokines and chemokine receptors is associated with chemotaxis and macrophage inflammatory proteins. From this cluster *CCR1 *and *CCR2 *are involved in monocyte chemotaxis [27]. A cluster associated with antigens contained Cluster Differentiation genes, and *CD14 *is a monocyte lineage specific marker. The immunologic receptor cluster contained a number of genes strongly associated with monocytes. One of these, *LILRB4 *(*ILT3*) is a cell surface molecule selectively expressed by the myeloid antigen presenting cells of the monocytic lineage [28]. As we did not find clusters characteristic for other myeloid cell-types, such as erythrocyte precursors, we postulate that AML patient group 5 is associated with precursor cells from the monocytic lineage.

In the original paper by Valk et al. [23] morphological characteristics of the leukemic cells were presented by means of the widely used 8 subtypes of the French-American-British (FAB) classification system. Using this classification we could verify whether our postulate is in concordance with the cells' appearance. In the study, patient group 5 contained specimens with FAB M4 or M5 subtypes. Specimens with an M4 classification contain cells that show granulocytic or monocytic maturation, and those with M5 have cells classified as monoblastic or monocytic.

Finally, we verified the presence of the mentioned genes and clusters in the other patient groups (Table 1). There is a considerable overlap with patient group 9, but not with other groups. According to the original paper, group 9 is indeed also composed of a mixture of the FAB classifications M4 and M5.

**Table 1:** Occurrence of monocyte specific clusters in patient groups.

	**Patient groups**										
**Cluster descriptions**	**3**	**4**	**5**	**6**	**7**	**8**	**9**	**10**	**12**	**13**	**16**

MHC 2	-	-	4↑	13↓	9↓	-	3↑	-	7↓	4↑	-
Cathepsins	-	11↓	9↑	-	3↓	-	-	4↓	3*	3↓	-
NADPH oxidase/respiratory burst	-	-	4↑	-	4↓	-	6↑	-	-	-	-

**Gene names**	**3**	**4**	**5**	**6**	**7**	**8**	**9**	**10**	**12**	**13**	**16**

CCR1	↓	↓	↑	-	-	-	↑	-	-	-	-
CCR2	↓	-	↑	-	-	-	↑	-	-	-	-
CD14	-	-	↑	-	-	-	↑	-	-	-	-
LILRB4	-	-	↑	-	-	-	-	-	-	-	↑

* The cathepsins of group 12 include 1 down-regulated and 2 up-regulated genes.

The upper half of the table shows for the patient groups the presence of the clusters of genes that were discussed for patient group 5. Several patient groups are not shown as the SAM analysis only yielded very few distinguishing genes. The size of the clusters is indicated and the arrows indicate if the genes are up- or down regulated. The lower half of the table shows the presence of the genes that were discussed in the text.

### DNA microarray dataset 2: Agonistic stimulation of the androgen receptor

In the second evaluation experiment on microarray data, we used Anni for the analysis of the list of 221 differentially expressed genes as measured with a DNA microarray following the agonistic stimulation of the androgen receptor in a prostate cancer cell line. The androgen receptor is a transcription factor, activated by the androgens testosterone and dihydrotestosterone and is responsible for development and maintenance of the function of the normal prostate and for growth of early stage prostatic cancer [29]. The complete annotation of the mentioned gene list is given in Additional file 3.

**Figure 2:**
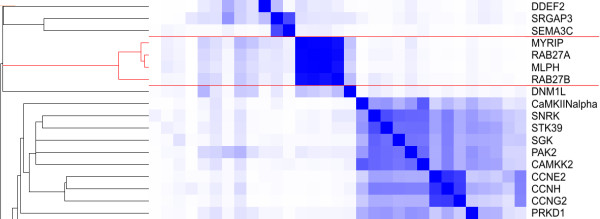
Fragment of the hierarchical clustering tree and heatmap based on the concept profiles for the genes differentially expressed following the agonistic stimulation of the androgen receptor. The tight cluster associated with melanosomes is highlighted.

The tightest cluster of genes consists of the genes *RAB27A*, *RAB27B*, *MYRIP *and *MLPH*, see Figure 2, and has an average cosine of 0,57, indicating a very strong within-cluster correlation. In Table 2 we show which concepts contribute the most to this average cosine score. The four gene concepts themselves are in the top of this list, which implies that these genes are regularly co-published. Other notable concepts are several myosin related concepts, the concepts melanosomes and melanocytes, and the concepts exocytosis and secretory vesicles. According to the MeSH vocabulary definitions: Myosin Type V is involved in organelle transport and membrane targeting. Melanosomes are melanin containing vesicles found in melanocytes and they are involved in skin pigmentation. The concepts exocytosis and secretory vesicles are both associated with the cellular release of material with membrane-limited vesicles. With a manual check of the literature linked by Anni to the four genes, we verified that the genes are indeed involved in the same process and their biological activity is in concord with the calculated annotation: all genes are associated with in the transport of melanosomes to the cell surface by interaction with myosin type V [30-32]. Certainly, there is no pigmentation in the prostate, but what quickly becomes apparent from literature is that these genes more generally deploy their activity in secretory lysosomes, of which melanosomes are only one example [33]. Secretory lysosomes are modified lysosomes that can proceed to regulated secretion in response to external stimuli, with a special role for *RAB27A *[30,33,34]. Terms associated with lysosomal processing are also part of the annotation, but are not shown in Table 2 since their contribution was below 0,5%.

**Table 2:** Concepts representative for the cluster RAB27B, MYRIP, MLPH, RAB27A as given by Anni.

		**Weight in concept profile**			
		
**Concept Name**	**Contribution (%)**	**RAB27B**	**MYRIP**	**MLPH**	**RAB27A**
RAB27A	52,17	0,61	0,74	0,73	1
MLPH	11.16	-	0,44	1	0,29
Myosin Type V	7,22	0,04	0,68	0,4	0,22
Melanosomes	6,7	0,12	0,3	0,47	0,27
RAB27B	4,06	1	0,14	-	0,11
MYRIP	2,98	0,07	1	0,09	0,06
Melanocytes	2,73	0,13	0,14	0,28	0,17
Myosins	2,33	0,04	0,38	0,22	0,12
Myosin Heavy Chains	1,72	-	0,46	0,18	0,09
GTP Phosphohydrolases	1,31	0,17	0,23	0,04	0,08
Actins	1,17	0,05	0,32	0,12	0,06
Exocytosis	0,87	0,08	0,12	0,08	0,12
Secretory Vesicles	0,68	0,07	0,16	0,06	0,09
Carrier Proteins	0,59	-	0,11	0,17	0,09
Organelles	0,54	0,11	-	0,12	0,09
rab GTP-Binding Proteins	0,52	0,16	-	0,04	0,12

In the first column the concept names are shown, in the second the percentage contribution of this concept to the average cosine score (0,57) of this group. We limited the number of concepts to a contribution of 0,5% to the average cosine score. The remaining columns show the weight of the concepts in the concept profiles of the genes whose names are shown in the column headings. These weights form the basis of the clustering of the 4 genes.

Secretory lysosomes may play their part in the major function of the prostate: the production and secretion of prostatic fluid. Several of the substances found in prostatic fluid point to a role for secretory lysosomes. Some of the secreted enzymes may be lysosomal; prostate acid phosphatase has for instance been localized in the lysosome [35]. Alternatively, *RAB27A *and associated proteins may be involved in the secretion of small vesicles called prostasomes. The latter hypothesis is supported by the identification of the *RAB27A *protein in prostasomes by proteome analysis [36]. It appears the potential roles of *RAB27A *and secretory lysosomes in the secretory processes of the prostate have currently not yet been investigated or reported. Semantic analysis of the literature associated with the genes differentially expressed in the microarray experiment, thus leads us to the novel hypothesis that secretory lysosomes are involved in the production of prostatic fluid and that their development and/or secretion are androgen-regulated processes.

## Discussion

We evaluated our concept profiling method in two steps. Firstly, we applied it to a controlled test set and compared its performance to that of our previously published ACS method [15,37]. The concept profiling method obtained high median scores for 4 of the 5 groups in the controlled test set, and performed significantly better than the ACS method for 2 groups, as well as overall. Secondly, we applied our method to actual research problems and annotated two DNA microarray datasets.

The first DNA microarray data set we analyzed, was the gene expression profile of the leukemic cells of a group of AML patients as identified in [23]. Little is known about the background of the leukemic cells in this cluster. With the Anni annotation and the underlying literature it was possible to identify several groups of genes and individual genes in the profile that indicate an association of the leukemic cells to cells of the monocytic lineage. This finding was in concordance with the morphological classification of the cells. The second data set consisted of a list of differentially expressed genes following the agonistic stimulation of the androgen receptor in a prostate cancer cell line. The Anni annotation revealed a cluster associated with, amongst others, melanosomes and secretory vesicles. Based on this finding and the underlying literature we formulated a hypothesis about the role of secretory lysosomes in prostate function. We conclude that Anni can be successfully used by molecular biologists studying DNA microarray datasets as a tool to automatically use the explicit and implicit information in literature.

The projected use of our method is the analysis of gene lists from high-throughput experiments. Our method is a useful addition to the current tool suite based on manual annotations or on automatic relation mining by analysis of the grammatical structure of sentences. Manual approaches, such as the GOA project, are limited in focus and tend to be incomplete due to the labor intensive annotation process. For example, in the case of the four melanosome-associated genes that we discussed, only *RAB27A *and *RAB27B *have, at the time of writing, a manual annotation by GOA. For these two genes the only curated annotation concerns their GTPase activity, even though there are numerous articles in Pubmed describing other features for which there are relevant Gene Ontology (GO) concepts, such as "melanosome". The computerized extraction of relations suffers from the limitation that the systems need to be trained to retrieve specific relations and entities. Hence, if the extraction algorithm is not trained for a specific relation it is likely to miss it. For example, the company Ariadne Genomics has constructed a relation database based on extensive natural language parsing (see e.g. [38]). They focused on the recognition of proteins and small molecules and their relationships. For both entities, at the time of writing, their database contains approximately 50,000 entries, but for biological processes there are only 263 entries which is a mere fraction of the more than 10,000 recognized in GO. The point is that the co-occurrence based method is simple and versatile. Associations can be retrieved between any two concepts once they can be recognized in text. Also the interpretation of associations differs from that of relationships. The association strengths in a concept profile for a concept A quantitatively reflect the statistical overrepresentation of concepts in texts in which concept A occurs. Hence, a concept profile of a particular concept can be seen as a view on the literature in which the concept is mentioned. This feature has value from an information retrieval point of view. The use of associations is also casting the net wide: not only are specific functional relationships retrieved, all significant associations between entities are retrieved, potentially even those not made explicit by the authors. This feature has been exploited for knowledge discovery purposes (see e.g. [39]).

Compared to other co-occurrence based approaches with similar objectives, our method may be considered an improvement on several points:

1. Anni was developed to be transparent, i.e. it is visible how the system comes to its associations. Transparency is a known problem with the ACS. The ACS was developed for knowledge discovery purposes and it uses an iterative algorithm to map concepts to a multi-dimensional space using concept co-occurrence data as input. In this space, the distance between concepts reflects the strength of one- and multi-step co-occurrence paths between the concepts. When applying the ACS, transparency was a problem for users of the system, as tracing distances between concepts back to the underlying literature was challenging. Compared to ACS, the Anni system is much more transparent: Anni provides a link to the underlying texts for every association between concepts. The system provides a coherence measure for a group of genes as well as the probability of a chance-occurrence of the group. Additionally, Anni illustrates the contribution of specific concepts to the coherence measure and shows the overlap between the concept profiles of the group members. It is, therefore, traceable why genes are clustered together. It is also trackable why certain concepts are associated with genes as the underlying articles can be accessed. In this aspect, Anni also contrasts favorably with, for instance, systems that use dimension reduction techniques [18-20]. Dimension reduction leaves the meaning of the dimensions unclear, and makes it difficult to verify, by consulting the underlying texts, whether the association between a gene and a dimension is true or relevant.

2. We used the controlled vocabulary Medical Subject Headings (MeSH) in addition to a gene thesaurus to identify concepts in texts. The use of thesauri allows the identification of multi-word concepts and the mapping of synonyms for the same concept, which reduces the noise caused by natural language variation. In addition, a thesaurus maps words or phrases to an abstract concept, thereby connecting it to all information available from other sources linked to this concept. For instance, a reference to a gene can be linked to its sequence or, as shown in this paper, semantic types can be used for filtering, and definitions of a concept can be used for interpretation. We used the semantic types associated with the biomedical concepts to focus the concept profiles on our area of interest. Several earlier approaches did not use a thesaurus for identifying biomedical concepts other than genes or proteins, e.g. [17]. The semantic filtering we used is more precise and adaptable than using different vocabularies as was done by [16].

3. The log-likelihood measure we use for the weighting of the associations between concepts is an important feature of our approach and has a sound statistical foundation. Some of the empirical approaches described in literature have properties that can be considered problematic. For example, Glenisson *et al. *[16] took the normalized inverse document frequency as the weight for a concept in a document. To produce the weight of a concept in a concept profile based on a selected set of documents, they averaged the concept's weight over the set. However, this procedure favors more frequently occurring concepts. Suppose two concepts in a large set of documents occur with rates *r*_1 _and *r*_2_, with *r*_1 _<*r*_2_, and thus for their weights will hold *w*_1 _> *w*_2 _in individual documents. When averaging the weights in a given subset of documents in which, say, both concepts occur with the same rates *r*_1_and *r*_2_, then the ratio of their original weights, w1w2
 MathType@MTEF@5@5@+=feaafiart1ev1aaatCvAUfKttLearuWrP9MDH5MBPbIqV92AaeXatLxBI9gBaebbnrfifHhDYfgasaacH8akY=wiFfYdH8Gipec8Eeeu0xXdbba9frFj0=OqFfea0dXdd9vqai=hGuQ8kuc9pgc9s8qqaq=dirpe0xb9q8qiLsFr0=vr0=vr0dc8meaabaqaciaacaGaaeqabaqabeGadaaakeaadaWcaaqaaiabdEha3naaBaaaleaacqaIXaqmaeqaaaGcbaGaem4DaC3aaSbaaSqaaiabikdaYaqabaaaaaaa@31EE@, will be reduced (by a factor r2r1
 MathType@MTEF@5@5@+=feaafiart1ev1aaatCvAUfKttLearuWrP9MDH5MBPbIqV92AaeXatLxBI9gBaebbnrfifHhDYfgasaacH8akY=wiFfYdH8Gipec8Eeeu0xXdbba9frFj0=OqFfea0dXdd9vqai=hGuQ8kuc9pgc9s8qqaq=dirpe0xb9q8qiLsFr0=vr0=vr0dc8meaabaqaciaacaGaaeqabaqabeGadaaakeaadaWcaaqaaiabdkhaYnaaBaaaleaacqaIYaGmaeqaaaGcbaGaemOCai3aaSbaaSqaaiabigdaXaqabaaaaaaa@31DA@) in the resulting concept profile. This may result in the weight of the more common concept becoming higher than that of the rarer concept.

Our approach had several limitations. Firstly, the thesaurus had to be curated for unnecessarily ambiguous concepts. We chose to do this in order to achieve a better precision, but, especially for genes, this will have reduced our retcall. Despite our curation efforts we encountered a small number of errors during our evaluation caused by polysemy, e.g. by gene symbols such as "protein s" as a synonym for the gene *PROS1*. More frequently we encountered errors in the thesaurus caused by errors in the underlying databases, such as "protein-tyrosine kinase" as a synonym for the gene *MUSK*. We expect our approach to further improve with a word-sense disambiguation module, as well as with progressive thesaurus curation. A second limitation in our study is the coverage of the thesaurus. New concepts arise constantly and may be very specifically used by a small group of specialists. Hence, to achieve optimal results for a thesaurus approach an up-to-date and domain-specific thesaurus is mandatory. A more flexible and dynamic approach to thesaurus construction is desirable. A third limitation is inherent in the use of co-occurrences to derive associations between concepts. Associations between concepts based on co-occurrences need not reflect actual biological relationships, even when their co-occurrence rate is far above the chance level.

## Conclusion

Anni was applied to a controlled dataset and to two DNA microarray datasets. We conclude that our method can be used to efficiently analyze a DNA microarray dataset based on both explicit and implicit information in the literature and expect that our system can be useful for the interpretation of high-throughput experiments.

## Methods

### Literature selection and indexing

We selected 2,585,901 abstracts with a Pubmed query for protein or gene mentioned together with mammals. MEDLINE titles, MeSH headings, and abstracts, if available, were indexed using Collexis software [40,41]. In this context, indexing means the identification of references to thesaurus concepts in text and mapping these references to the concepts. Prior to indexing we removed stop words. All words are mapped to the uninfiected form produced by the normalizer of the lexical variant generator [42]. The thesaurus we used for indexing was composed of two parts: MeSH and a human gene thesaurus derived from multiple databases [43]. For MeSH we used the UMLS semantic types [44] to select concepts that convey relevant biological information about genes. The filter was developed by molecular biologists and the selected semantic types are given in Additional file 4. This filtering facilitated the interpretation of the profiles and also slightly increased performance on our test set (data not shown). The gene thesaurus was expanded by rewrite rules to take into account common spelling variations [45]. For instance, numbers were replaced with roman numerals and vice versa, and hyphens before numbers at the end of gene symbols were inserted or removed (e.g. "WAF1" was rewritten as "WAF-1" and added as a synonym). Then, potentially highly ambiguous terms (less than five characters, none of them a digit) were removed in order to obtain a high precision on gene recognition. Gene symbols or full gene names that refer to more than one gene in the thesaurus were rejected as well.

### ACS

The ACS algorithm has been described in detail before [37] and was developed to be applied for knowledge discovery. Briefly, it is a Hebbian-type of learning algorithm that in an iterative process positions the thesaurus concepts in a multidimensional Euclidean space. In this space the dimensions do not take a specific meaning, but just allow the positioning of the concepts relative to each other. The position of a concept follows from the mapping of co-occurrence relations (paths) between concepts to distances. A distance between two concepts will not only reflect the co-occurrence of the two concepts, a one-step relation, but also indirect, multi-step relations between the two concepts. As the distance between concepts reflects the strength of both one- and multi-step co-occurrence paths between the concepts, it is possible that concepts are placed close to each other that do not have a direct co-occurrence. The idea behind the ACS is that we may postulate in such a case that there is an actual association between these concepts, which has not been reported in literature.

For the construction of the ACS we used a selection of literature. For the test set for each gene a maximum of 1000 randomly selected abstracts mentioning the gene are included. For the ACS we used a vector format to represent documents with term frequency * inverse document frequency weighting and standard algorithm settings [15].

### Concept-profile generation

A concept profile of gene *i *is an M-dimensional vector **w**_**i **_= (*w*_*i*1_, *w*_*i*2_,..., *w*_*iM*_) where M is the number of concepts in the thesaurus. The weight *w*_*ij *_for a concept *j *in this profile indicates the strength of its association to the concept *i*. The weights in a concept profile for concept *i *are derived from the set of documents in which concept *i *occurs. To obtain *w*_*ij *_we employ the log likelihood ratio measure [22]. Two hypotheses are used: 1. The probability of occurrence of concept *j *is independent of the occurrence of concept *i*; 2. The probability of occurrence of concept *j *is dependent of the occurrence of concept *i*. For each hypothesis a likelihood is calculated based on the observed data using the binomial distribution. The ratio of these likelihoods tells us how much more likely one hypothesis is over the other, or, in other words, how sure we are that there is a dependency. A feature of the log likelihood ratio is that it behaves relatively well for sparse data [21], which is an advantage in our case.

The following equations give the likelihood ratio *λ *of concepts *i *and j:

λ(i,j)=L(nij,ni,p)L(nj−nij,N−ni,p)L(nij,ni,p1)L(nj−nij,N−ni,p2)
 MathType@MTEF@5@5@+=feaafiart1ev1aaatCvAUfKttLearuWrP9MDH5MBPbIqV92AaeXatLxBI9gBaebbnrfifHhDYfgasaacH8akY=wiFfYdH8Gipec8Eeeu0xXdbba9frFj0=OqFfea0dXdd9vqai=hGuQ8kuc9pgc9s8qqaq=dirpe0xb9q8qiLsFr0=vr0=vr0dc8meaabaqaciaacaGaaeqabaqabeGadaaakeaaiiGacqWF7oaBcqGGOaakcqWGPbqAcqGGSaalcqWGQbGAcqGGPaqkcqGH9aqpdaWcaaqaaiabdYeamjabcIcaOiabd6gaUnaaBaaaleaacqWGPbqAcqWGQbGAaeqaaOGaeiilaWIaemOBa42aaSbaaSqaaiabdMgaPbqabaGccqGGSaalcqWGWbaCcqGGPaqkcqWGmbatcqGGOaakcqWGUbGBdaWgaaWcbaGaemOAaOgabeaakiabgkHiTiabd6gaUnaaBaaaleaacqWGPbqAcqWGQbGAaeqaaOGaeiilaWIaemOta4KaeyOeI0IaemOBa42aaSbaaSqaaiabdMgaPbqabaGccqGGSaalcqWGWbaCcqGGPaqkaeaacqWGmbatcqGGOaakcqWGUbGBdaWgaaWcbaGaemyAaKMaemOAaOgabeaakiabcYcaSiabd6gaUnaaBaaaleaacqWGPbqAaeqaaOGaeiilaWIaemiCaa3aaSbaaSqaaiabigdaXaqabaGccqGGPaqkcqWGmbatcqGGOaakcqWGUbGBdaWgaaWcbaGaemOAaOgabeaakiabgkHiTiabd6gaUnaaBaaaleaacqWGPbqAcqWGQbGAaeqaaOGaeiilaWIaemOta4KaeyOeI0IaemOBa42aaSbaaSqaaiabdMgaPbqabaGccqGGSaalcqWGWbaCdaWgaaWcbaGaeGOmaidabeaakiabcMcaPaaaaaa@7817@

with *n*_*i *_and *n*_*j *_the number of documents in which concepts *i *and *j *occur, *n*_*ij *_the number of documents in which both concepts occur, *N *is the number of documents in the corpus, p=niN,p1=nijni,p2=nj−nijN−ni
 MathType@MTEF@5@5@+=feaafiart1ev1aaatCvAUfKttLearuWrP9MDH5MBPbIqV92AaeXatLxBI9gBaebbnrfifHhDYfgasaacH8akY=wiFfYdH8Gipec8Eeeu0xXdbba9frFj0=OqFfea0dXdd9vqai=hGuQ8kuc9pgc9s8qqaq=dirpe0xb9q8qiLsFr0=vr0=vr0dc8meaabaqaciaacaGaaeqabaqabeGadaaakeaacqWGWbaCcqGH9aqpdaWcaaqaaiabd6gaUnaaBaaaleaacqWGPbqAaeqaaaGcbaGaemOta4eaaiabcYcaSiabdchaWnaaBaaaleaacqaIXaqmaeqaaOGaeyypa0ZaaSaaaeaacqWGUbGBdaWgaaWcbaGaemyAaKMaemOAaOgabeaaaOqaaiabd6gaUnaaBaaaleaacqWGPbqAaeqaaaaakiabcYcaSiabdchaWnaaBaaaleaacqaIYaGmaeqaaOGaeyypa0ZaaSaaaeaacqWGUbGBdaWgaaWcbaGaemOAaOgabeaakiabgkHiTiabd6gaUnaaBaaaleaacqWGPbqAcqWGQbGAaeqaaaGcbaGaemOta4KaeyOeI0IaemOBa42aaSbaaSqaaiabdMgaPbqabaaaaaaa@50D1@, and *L*(*k*,*l*,*x*) = *x*^*k*^(1 - *x*)^*l*-*k*^. A feature of likelihood ratios is that -2 times the log of the likelihood ratio is asymptotically *χ*^2 ^distributed [22], which can be used to test whether there is a statistically significant divergence from independence. The weight of concept *j *in the concept profile of concept *i *is given by:

wij=log⁡λ(i,j)L
 MathType@MTEF@5@5@+=feaafiart1ev1aaatCvAUfKttLearuWrP9MDH5MBPbIqV92AaeXatLxBI9gBaebbnrfifHhDYfgasaacH8akY=wiFfYdH8Gipec8Eeeu0xXdbba9frFj0=OqFfea0dXdd9vqai=hGuQ8kuc9pgc9s8qqaq=dirpe0xb9q8qiLsFr0=vr0=vr0dc8meaabaqaciaacaGaaeqabaqabeGadaaakeaacqWG3bWDdaWgaaWcbaGaemyAaKMaemOAaOgabeaakiabg2da9maalaaabaGagiiBaWMaei4Ba8Maei4zaCgcciGae83UdWMaeiikaGIaemyAaKMaeiilaWIaemOAaOMaeiykaKcabaGaemitaWeaaaaa@3E6B@

*L *is the theoretical maximum score of log *λ*, which is obtained when a concept always and only occurs together with concept *i*. This factor normalizes for the effects of the occurrence rate of concept *i*, which is convenient when comparing weights between profiles.

For every concept co-occurring with concept *i *we calculated the log likelihood ratio, but in order for a concept to be included in the concept profile the null hypothesis (the occurrence of *j *is independent of the occurrence of *i*) has to be rejected at a significance level of 0,005. For efficiency reasons we included only the most significant concepts to a maximum of 200 concepts.

Associations between concepts are calculated based on concept profiles using cosine similarity scores [46].

### The Anni system

In order to analyze a list of genes by means of their concept profiles we developed 'Anni'. The tool retrieves and displays the concept profile of a gene and can also characterize any combination of genes. The components of the Anni system are two databases and a web-based graphical user interface. The first database contains concept profiles for human genes. The second database contains the indexed literature underlying the concept profiles, which is used in the system to identify the documents supporting the associations in a concept profile. The interface provides the following functionality: 1. The user can specify a list of genes to analyze based Affymetrix, Entrez Gene or Swiss-Prot identifiers: 2. Groups of genes with similar profiles can be found using hierarchical clustering. As the input for the clustering algorithm, we use for each gene in the input list, the cosine scores between the concept profiles of this gene and the other genes. We used mean linkage hierarchical clustering with cosine as similarity metric: 3. An identified cluster of genes is given a coherence measure, the average of the cosine scores of all possible pairs within the cluster. To assess the significance of the average cosine score we give the probability that the same score or higher would be found in a randomly formed group of the same size. This probability was determined from the distribution of scores from a 10000-fold random sampling of groups of gene profiles; 4. A cluster of genes is characterized by showing the relative contribution of individual concepts as a percentage. In addition the weights of these concepts in the concept profiles are shown, which facilitates an easy assessment of the similarity of the profiles; 5. For every association in a concept profile a link to the underlying literature is provided.

For clarity, the only overlap between the Anni system and the ACS is the underlying database of indexed documents and the used thesaurus. Apart from this, the systems share no methodology.

To analyze gene lists in a standardized manner we used the following protocol. All clusters with a cosine coefficient greater than 0,15 and containing at least three genes were analyzed. The probability that the average cosine score was found by chance should be < 0,005. A cluster may be split into smaller, more consistent clusters, if there are smaller clusters with distinct common functions.

### Evaluation

For comparison of the ACS and the concept profile method we used the test set and the evaluation procedure as described in [15]. The test set was made by pooling five groups of genes that share a biological relationship. Each group represented a different aspect of gene biology, being function, organelle, biological process, metabolic pathway, or association with a disease. Only human genes were taken into consideration. The selected groups are: spermatogenesis, 15 genes: lysosome; 10 genes; chaperone activity, 7 genes: breast cancer, 9 genes: glycolysis, 6 genes. For the evaluation, both the ACS and the concept profile method were employed to produce a ranking of the set of genes relative to one so-called seed gene. All genes in turn served as a seed, producing a ranking for each of the other 46 genes in our set. For the concept profile method, genes were rank-ordered according to the cosine similarity scores [46] between the concept profile vector of the genes and the seed gene. Ties were ordered randomly. For the ACS, genes from the set were rank-ordered according to their Euclidean distances to the seed gene. For each gene a receiver operating characteristics (ROC) curve was then constructed [47]. The area under the curve (AUC) was used as a performance measure [48]. This value varies between 0 and 1. An AUC of 1 represents perfect ordering, i.e. all genes belonging to the group of the seed gene are at the top of the list followed by the other genes. The AUC has the useful property that a value of 0,5 represents random ordering [48]. This property provides us, in a way, with a built-in negative control.

To determine whether the AUC scores differed significantly between the two methods, we used the non-parametric Wilcoxon signed ranks test. The test requires the AUC scores of the genes to be independent. Because this is not true in this case, we applied bootstrapping [49] to estimate the distribution of the Wilcoxon test statistic. We generated 100 new sets of genes by sampling genes from the original set with replacement. The sampling was stratified over the five gene groups to obtain groups of equal size as in the original set. AUCs were calculated for both methods, and the Wilcoxon signed ranks test was applied to measure the difference between the two methods per gene group. The results obtained for the 100 sets were used to determine if the two methods differ in performance at the 0,05 level.

### Description DNA microarray data sets

The first set consisted of data from a recent study about prognostically useful gene-expression profiles in AML [23]. Gene expression in leukemic blast cells from 285 patients was measured. Clustering of the gene expression data resulted in 16 groups of patients with distinct profiles. For each cluster a profile of genes with the most distinguishing gene expression patterns was made with the significance analysis of microarray (SAM) method. For our analysis genes with a SAM score higher than 4 or lower than -4 were selected. Data acquisition and processing are described in detail in the original paper.

The second set consisted of differentially expressed genes following the agonistic stimulation of the androgen receptor in a prostate cancer cells. The androgen-dependent LNCaP prostate cancer cell line was maintained in RPMI media with 5% fetal calf serum and penicillin/streptomycin (Invitrogen, Merelbeke, Belgium). Before R1881 treatment, cells were androgen-deprived for 72 hours in a medium containing 5% dextran-filtered, charcoal-stripped fetal calf serum. After androgen deprivation, the medium was supplemented for 2, 4, 6 or 8 hours with 1 nM synthetic androgen R1881 or ethanol vehicle as the control. Three *μg *of total RNA was used for a T7 based linear mRNA amplification protocol [50]. Two micrograms of amplified RNA were used to produce Cy3- or Cy5-labeled cDNA. cDNAs from R1881-treated and control cells were compared directly by hybridization to the same microarray. This was done in duplicate with reversed Cy dye labeling. The cDNA microarrays were manufactured at the Central Microarray Facility of the Netherlands Cancer Institute (NKI, Amsterdam, The Netherlands) and contained over 18,000 features that have been selected from the Research Genetics Human Sequence Verified Library (Invitrogen). Normalization of spot intensities was performed using R-routines (Lowess method) using the NKI Microarray Normalization Tools . Genes were considered to be up or down-regulated by R1881 when both dye swaps gave a ratio larger than 1,62 (2log 0,7) for at least one time point. The data have been deposited in NCBIs Gene Expression Omnibus [51]and are accessible through GEO Series accession number GSE4027 and GSE1159.

## List of abbreviations used

**ACS **Associative Concept Space

**AML **Acute Myeloid Leukemia

**AUC **Area Under the Curve

**FAB **French-American-British classification system

**GO **Gene Ontology

**GOA **Gene Ontology Annotation project

**MeSH **Medical Subject Headings

**MHC-II **Major Histocompatibility Complex class 2

**ROC-curve **Receiver Operating Characteristics curve

**UMLS **Unified Medical Language System

## Authors' contributions

RJ conceived of the methodology, performed the experiments and wrote the manuscript. GJ and LD supervised and contributed to the development of Anni and together with PH analyzed dataset 2 and contributed to the manuscript. BW and RD participated in the analysis of dataset 1. BM was involved in drafting and critically revising the manuscript. JK conceived of the study and supervised the experiments and writing the manuscript. All authors read and approved the final manuscript.

## Supplementary Material

Additional File 1 The controlled test set. Click here for file

Additional File 2 Annotation of the first DNA microarray dataset. Click here for file

Additional File 3 Annotation of the second DNA microarray dataset. Click here for file

Additional File 4 Semantic types used for filtering. Click here for file
