# Supplementary material for: Text-derived concept profiles support assessment of DNA microarray data for acute myeloid leukemia and for androgen receptor stimulation
Source: BMC Bioinformatics. 2007 Jan 18;8:14. doi: 10.1186/1471-2105-8-14 (PMC1784107; doi:10.1186/1471-2105-8-14)
Supplement: Additional File 1 — The controlled test set. [file 1471-2105-8-14-S1.rtf]

Appendix 1, 
Test set.

Table 1, Genes included in the test set used for the evaluation of the ACS and concept profiling method. The test set does not include the genes with erroneous annotations as described in Jelier et al. (2005).
Entrez Gene ID	Gene Symbol	Gene Name	Group	
325	APCS	serum amyloid P component	Chaperone	
3998	LMAN1	mannose-binding lectin 1	Chaperone	
6102	RP2	retinitis pigmentosa 2	Chaperone	
6687	SPG7	spastic paraplegia 7	Chaperone	
6950	TCP1	t-complex 1	Chaperone	
7249	TSC2	tuberous sclerosis 2	Chaperone	
11140	CDC37	cell division cycle 37 homolog (S. cerevisiae)	Chaperone	
410	ARSA	arylsulfatase A	Lysosome	
411	ARSB	arylsulfatase B	Lysosome	
412	STS	steroid sulfatase	Lysosome	
1200	CLN2	neuronal ceroid-lipofuscinosis 2	Lysosome	
2548	GAA	acid alpha-glucosidase	Lysosome	
2581	GALC	galactosylceramidase	Lysosome	
3916	LAMP1	lysosomal-associated membrane protein 1	Lysosome	
4353	MPO	myeloperoxidase	Lysosome	
4758	NEU1	sialidase 1	Lysosome	
8692	HYAL2	hyaluronoglucosaminidase 2	Lysosome	
2302	FOXJ1	forkhead box J1	Spermatogenesis	
2492	FSHR	follicle stimulating hormone receptor	Spermatogenesis	
2649	NR6A1	nuclear receptor subfamily 6, group A, member 1	Spermatogenesis	
3010	HIST1H1T	histone 1, H1t	Spermatogenesis	
3206	HOXA10	homeo box A10	Spermatogenesis	
3640	INSL3	insulin-like 3	Spermatogenesis	
5619	PRM1	protamine 1	Spermatogenesis	
5620	PRM2	protamine 2	Spermatogenesis	
6046	BRD2	bromodomain containing 2	Spermatogenesis	
6847	SYCP1	synaptonemal complex protein 1	Spermatogenesis	
8287	USP9Y	ubiquitin specific protease 9, Y chromosome	Spermatogenesis	
8607	RUVBL1	RuvB-like 1 (E.coli)	Spermatogenesis	
8900	CCNA1	cyclin A1	Spermatogenesis	
9191	DEDD	death effector domain containing	Spermatogenesis	
23626	SPO11	sporulation protein, meiosis-specific, SPO11 homolog (S. cerevisiae)	Spermatogenesis	
672	BRCA1	breast cancer 1, early onset	Breast Cancer	
675	BRCA2	breast cancer 2, early onset	Breast Cancer	
1956	EGFR	epidermal growth factor receptor	Breast Cancer	
2064	ERBB2	erythroblastic leukemia viral oncogene homolog 2	Breast Cancer	
2066	ERBB4	erythroblastic leukemia viral oncogene homolog 4 	Breast Cancer	
2099	ESR1	estrogen receptor 1	Breast Cancer	
5241	PGR	progesterone receptor	Breast Cancer	
5915	RARB	retinoic acid receptor, beta	Breast Cancer	
7157	TP53	tumor protein p53	Breast Cancer	
226	ALDOA	fructose-bisphosphate aldolase A	Glycolysis	
2023	ENO1	enolase 1	Glycolysis	
2597	GAPD	glyceraldehyde-3-phosphate dehydrogenase	Glycolysis	
2821	GPI	glucose phosphate isomerase	Glycolysis	
5230	PGK1	phosphoglycerate kinase 1	Glycolysis	
5236	PGM1	phosphoglucomutase 1	Glycolysis	
