# Supplementary material for: Text-derived concept profiles support assessment of DNA microarray data for acute myeloid leukemia and for androgen receptor stimulation
Source: BMC Bioinformatics. 2007 Jan 18;8:14. doi: 10.1186/1471-2105-8-14 (PMC1784107; doi:10.1186/1471-2105-8-14)
Supplement: Additional File 2 — Annotation of the first DNA microarray dataset. [file 1471-2105-8-14-S2.rtf]

Appendix 2
Anni annotation of the gene expression profile of patient group 5

Genes in analysis: 990
In the annotations concepts are shown if their percentage contribution to the average cosine score for the group exceeds 0.5%. 
Summary of identified clusters 
 Cluster description:	# of genes	
Antigens	8	
Apoptose	17	
Calcium binding proteints (S100-family)	6	
Cathepsins	9	
Cell cycle	13	
Chemokines and chemokine receptors	8	
Colorectal cancer	5	
Cytochrome P-450 and liver microsomes	6	
Fc receptors and immunoglobin G, cluster 1 	3	
Fc receptors and immunoglobin G, cluster 2	3	
Fluorouracil	4	
Galactosyltransferases/N-Acetylglucosaminyltransferases	5	
Golgi apparatus	4	
GTPase-Activating Proteins	6	
Guanine Nucleotide exchange factors	6	
Hexoseaminidases and gangliosidoses	3	
HLA proteins, MHC1	4	
HLA proteins, MHC2	4	
Immunologic Receptors	9	
Insulin like growth factor receptor 1 	3	
LDL receptor	5	
LeukoTrienes	5	
Ligases	5	
Matrix Metalloproteinases	4	
Membrane glycoproteins and cell surface receptors	12	
Metallothionein	4	
MicroFilament and Actins	20	
Multiple/neoplasm-Drug Resistance	4	
NADPH oxidase/ phagocytes/neutrophils	4	
Oxidoreductases	3	
Phosphotransferase, cluster 1	5	
Phosphotransferase, cluster 2	25	
Proteoglycans and Basement Membrane	7	
Purinergic receptors	3	
Ribosomal proteins	11	
Sulfatases	3	
Transcription factors and nuclear proteins	58	
Transferrin	4	
Tumor necrosis factor	11	
Vesicular protein transport and exocytosis	3	
Annicluster 1
Concept	Contribution %	SGSH	GNS	STS	
Sulfatases	39.35	0.11	0.23	0.52	
STS	13.24	0	0.06	1	
Arylsulfatases	10.25	0.06	0.09	0.31	
Mucopolysaccharidosis III	9.81	0.67	0.08	0	
Mucopolysaccharidoses	9.02	0.52	0.09	0	
Heparitin Sulfate	3.33	0.28	0.06	0	
Hydrolases	2.35	0.37	0.03	0	
ARSA	1.86	0.05	0.14	0.02	
Cerebroside-Sulfatase	1.62	0.04	0.13	0.01	
N-Acetylgalactosamine-4-Sulfatase	1.4	0.05	0.11	0.01	
Sulfates	1.29	0.02	0.07	0.06	
Ichthyosis	1.18	0	0.02	0.23	
Enzymes	0.94	0.05	0.04	0.03	
Glycosaminoglycans	0.9	0.1	0.05	0	
MPS III A	0.85	0.09	0.05	0	
GNS	0.82	0	1	0	
Lysosomal Storage Diseases	0.52	0.12	0.02	0	
AnniCluster 2 
Concept	Contribution %	LTB4R	LTA4H	ALOX5AP	ALOX5	MGST2	
Arachidonate 5-Lipoxygenase	18.2	0.03	0.23	0.45	0.79	0	
Leukotrienes	17.52	0.05	0.41	0.39	0.27	0.19	
ALOX5	14.78	0.01	0.16	0.32	1	0	
LTA4H	11.54	0	1	0.06	0.02	0.3	
Leukotriene B4	9.29	0.33	0.31	0.12	0.18	0	
ALOX5AP	8.49	0	0.09	1	0.06	0.13	
Leukotriene C4	4.04	0	0.26	0.12	0.06	0.21	
Leukotriene A4	2.36	0	0.36	0.05	0.03	0.12	
Arachidonic Acids	2.26	0.01	0.2	0.12	0.17	0	
Arachidonic Acid	2.26	0.01	0.2	0.12	0.17	0	
Receptors, Leukotriene	1.27	0.36	0.03	0.02	0.02	0.05	
Lipoxygenase Inhibitors	1.21	0.01	0.04	0.17	0.11	0.04	
Glutathione Transferase	1.1	0	0.09	0.03	0	0.32	
MGST2	0.62	0	0.02	0.01	0	1	
AnniCluster 3
Concept	Contribution %	NAGA	GM2A	HEXB	
beta-N-Acetylhexosaminidase	43.9	0.35	0.12	0.56	
HEXB	17.63	0	0.12	1	
Hexosaminidases	17.53	0.36	0.03	0.29	
HEXA	8.88	0	0.15	0.39	
Gangliosidoses	2.71	0	0.17	0.11	
Sandhoff Disease	2.21	0	0.05	0.28	
Tay-Sachs Disease	1.71	0	0.1	0.11	
GM2A	1.39	0	1	0.01	
Gangliosides	1.01	0	0.11	0.06	
Lysosomal Storage Diseases	0.6	0.04	0.04	0.03	
Lysosomes	0.57	0.02	0.05	0.04	
AnniCluster 4
Concept	Contribution %	NAGPA	EXTL2	MGAT1	OGT	MGEA5	
N-Acetylglucosaminyltransferases	25.63	0.03	0.41	0.38	0.35	0.11	
A4GALT	25.38	0.03	0.41	0.38	0.35	0.11	
OGT	17.49	0	0.14	0.01	1	0.2	
Acetylglucosamine	10.92	0.13	0.08	0.05	0.18	0.36	
FLJ20277	5.19	0.09	0.06	0.12	0.18	0.08	
Enzymes	3.35	0.16	0	0.12	0.08	0.07	
MGEA5	2.33	0	0	0	0.05	1	
Oligosaccharides	1.62	0.14	0	0.06	0.12	0	
Glycosyltransferases	1.14	0	0.1	0.09	0.09	0	
Mannose	0.86	0.23	0	0.04	0.02	0	
AnniCluster 5
Up-up-down
Concept	Contribution %	EHD1	EHD4	EHD3	
EHD1	48.54	1	0.48	0.78	
EHD3	17.76	0.1	0.6	1	
EHD4	15.98	0.06	1	0.6	
EPS15	10.33	0.13	0.41	0.68	
Carrier Proteins	1.85	0.03	0.25	0.25	
Vesicular Protein Transport	1.56	0.07	0.11	0.27	
Gene Expression	0.6	0.02	0.1	0.21	
Cytoplasmic Vesicles	0.56	0.01	0.14	0.14	
Heart	0.51	0.02	0.13	0.13	
AnniCluster 6
Concept	Contribution %	IGFBP2	IGF1R	DEGS	
IGF1	26.31	0.23	0.46	0.2	
Insulin-Like Growth Factor I	20.28	0.2	0.39	0.18	
Receptor, IGF Type 1	9.38	0.03	0.75	0.08	
Insulin-Like Growth-Factor-Binding Proteins	8.55	0.31	0.05	0.16	
Somatomedins	6.63	0.18	0.15	0.1	
IGF1R	5.93	0.02	1	0.04	
IGFBP2	5.63	1	0.01	0.03	
Insulin-Like Growth Factor-Binding Protein 2	2.72	0.5	0.01	0.04	
Insulin-Like Growth Factor II	2.7	0.13	0.07	0.08	
DEGS	1.86	0.01	0.01	1	
IGFBP3	1.84	0.23	0.02	0.05	
IGF2	1.36	0.1	0.05	0.05	
IGFBP1	1.08	0.18	0.02	0.03	
Insulin-Like Growth Factor Binding Protein 3	0.96	0.17	0.01	0.03	
Receptors, Somatomedin	0.6	0.02	0.14	0.02	
INSL3	0.53	0.01	0.01	0.25	
AnniCluster 7
DOUBTFULL? Homonyms?
Concept	Contribution %	NF1	JJAZ1	CENTA2	EVI2B	EVI2A	
NF1	23.01	1	0.16	0.36	0.44	0.3	
EVI2A	17.17	0.01	0.15	0.36	0.54	1	
EVI2B	12.09	0.01	0.16	0.38	1	0.29	
JJAZ1	9.13	0	1	0.38	0.19	0.09	
Neurofibromatosis 1	9.08	0.52	0.12	0.22	0.33	0.2	
Genes, Neurofibromatosis 1	3.87	0.27	0.06	0.25	0.22	0.12	
CENTA2	3.62	0	0.12	1	0.14	0.07	
Chromosomes, Human, Pair 17	3.51	0.06	0.12	0.29	0.23	0.2	
Neurofibromin 1	2.78	0.34	0.06	0.11	0.17	0.1	
HCA66	2.29	0	0.13	0.48	0.16	0.08	
FLJ12735	2.29	0	0.13	0.48	0.16	0.08	
FLJ22729	2.29	0	0.13	0.48	0.16	0.08	
CRLF3	2.29	0	0.13	0.48	0.16	0.08	
Neurofibroma	1.48	0.09	0.06	0.24	0.16	0.05	
OMG	0.97	0	0.05	0.17	0.19	0.09	
HSA272196	0.57	0	0.07	0.23	0.08	0.04	
RAB11-FIP4	0.57	0	0.07	0.23	0.08	0.04	
RNF135	0.57	0	0.07	0.23	0.08	0.04	
AnniCluster 8
Resp burst: up
Concept	Contribution %	CYBB	NCF2	NCF1	CYBA	
NADPH Oxidase	20.46	0.29	0.54	0.52	0.23	
CYBB	18.33	1	0.27	0.2	0.18	
NCF2	12.2	0.08	1	0.35	0.06	
Granulomatous Disease, Chronic	8.65	0.73	0.13	0.11	0.19	
NCF1	8.45	0.05	0.32	1	0	
Superoxides	6.7	0.15	0.29	0.24	0.2	
Oxidase	5.58	0.12	0.3	0.23	0.17	
Phosphoproteins	5.22	0.04	0.27	0.29	0.2	
NADPH Dehydrogenase	2.26	0.03	0.12	0.08	0.35	
Phagocytes	1.98	0.13	0.16	0.12	0.08	
Neutrophils	1.76	0.14	0.17	0.14	0.03	
MTCYB	1.18	0.1	0.11	0.05	0.1	
Cytochrome b	1.18	0.1	0.11	0.05	0.1	
Respiratory Burst	0.84	0.08	0.1	0.1	0.04	
NADH	0.81	0.07	0.13	0.09	0.03	
DECR1	0.79	0.07	0.15	0.11	0	
DNCH1	0.7	0.04	0.1	0.01	0.16	
AnniCluster 9
Up xcept HSPG2
Concept	Contribution %	PRG1	CSPG2	SDC4	HSPG2	HPSE	NID	C1orf38	
Proteoglycans	25.27	0.38	0.4	0.34	0.3	0.06	0.07	0	
SDC2	11	0.02	0.04	0.26	0.5	0.17	0.11	0	
Heparan Sulfate Proteoglycan	10.18	0.02	0.04	0.22	0.5	0.17	0.11	0	
Basement Membrane	9.69	0	0.01	0	0.23	0.08	0.35	0.35	
Heparitin Sulfate	8.26	0.05	0.02	0.19	0.32	0.3	0.05	0	
NID	6.12	0	0	0	0.06	0	1	0.15	
Laminin	4.53	0	0.01	0	0.12	0.01	0.43	0.2	
HSPG2	4.26	0	0.06	0.02	1	0.01	0.07	0	
Core Protein	3.3	0.15	0.09	0.15	0.15	0.01	0.01	0	
Extracellular Matrix	2.94	0.01	0.13	0.02	0.1	0.14	0.14	0	
Glycosaminoglycans	2.06	0.12	0.09	0.1	0.09	0.04	0.01	0	
Proteochondroitin Sulfate	1.75	0.07	0.43	0	0.06	0	0.01	0	
Proteochondroitin Sulfates	1.75	0.07	0.43	0	0.06	0	0.01	0	
CSPG2	1.51	0	1	0.01	0.05	0	0	0	
Collagen Type IV	0.85	0	0	0	0.05	0	0.16	0.1	
BGN	0.67	0.06	0.19	0.01	0.04	0	0	0	
Membrane Glycoproteins	0.58	0	0	0.09	0.02	0	0.17	0	
Chondroitin Sulfates	0.55	0.07	0.08	0.03	0.04	0.01	0.01	0	
AnniCluster 10
All up: associated with monocytes
Concept	Contribution %	HM74	CCL3	CCL23	CCL20	CCR5	CCR1	CCR2	CX3CR1	
Receptors, Chemokine	26.71	0.19	0.09	0.2	0.37	0.35	0.54	0.57	0.29	
Chemokines	21.9	0.1	0.32	0.28	0.41	0.18	0.37	0.3	0.35	
CCR5	12.17	0.02	0.05	0.04	0.01	1	0.37	0.37	0.11	
CCR1	5.71	0.05	0.04	0.07	0.02	0.07	1	0.2	0.07	
CCR2	5.36	0.01	0.02	0	0.03	0.1	0.28	1	0.08	
Macrophage Inflammatory Proteins	2.76	0	0.11	0.14	0.4	0.05	0.13	0.05	0.01	
CCL3	2.28	0	1	0.06	0.02	0.02	0.09	0.02	0.01	
CCL5	2.28	0.02	0.19	0.14	0.01	0.1	0.19	0.07	0.03	
CXCR4	2.13	0.07	0.01	0.04	0.01	0.3	0.13	0.12	0.07	
RANTES	1.95	0.02	0.17	0.14	0.01	0.09	0.17	0.07	0.03	
CCL20	1.63	0	0	0.15	1	0	0.01	0.01	0	
Macrophage Inflammatory Protein-1	1.23	0	0.37	0.07	0	0.06	0.11	0.03	0.01	
CCR3	1.21	0.01	0.01	0.04	0.01	0.08	0.24	0.15	0.07	
CCR6	0.95	0	0	0.14	0.34	0.01	0.05	0.03	0.01	
Chemotaxis	0.93	0.02	0.04	0.13	0.07	0.02	0.09	0.06	0.06	
Receptors, Cytokine	0.78	0.11	0.02	0	0	0.03	0.05	0.08	0.17	
Monocyte Chemoattractant Protein-1	0.68	0	0.1	0.03	0.01	0.02	0.06	0.22	0.02	
CCL23	0.66	0	0	1	0.05	0	0.01	0	0	
CX3CR1	0.59	0	0	0.01	0	0.01	0.02	0.02	1	
T-Lymphocytes	0.55	0.03	0.04	0.04	0.06	0.08	0.06	0.05	0.02	
AnniCluster 11
Up down up
Concept	Contribution %	DHRS9	AKR1C3	HADHSC	
Hydroxysteroid Dehydrogenases	19.48	0.48	0.36	0.12	
DHRS9	17.07	1	0.12	0.1	
Oxidoreductases	16.06	0.26	0.23	0.32	
AKR1C3	8.57	0.13	1	0	
AKR1C2	4.86	0.26	0.28	0	
HSD17B3	4.46	0.23	0.29	0	
3-Hydroxysteroid Dehydrogenases	4.21	0.22	0.28	0	
Steroids	3.45	0.26	0.11	0.06	
AKR1C4	3.36	0.13	0.37	0	
AKR1C1	3.13	0.17	0.27	0	
Hydroxysteroids	2.23	0.16	0.2	0	
HADHSC	1.88	0.03	0	1	
17-Hydroxysteroid Dehydrogenases	1.18	0.12	0.04	0.07	
3alpha-Androstanediol	1.17	0.18	0.09	0	
DHDH	0.91	0.09	0.16	0	
Androstanes	0.89	0.1	0.04	0.06	
Ketosteroids	0.81	0.14	0.09	0	
Stanolone	0.69	0.16	0.06	0	
20-Hydroxysteroid Dehydrogenases	0.67	0.13	0.08	0	
Androstane-3,17-diol	0.53	0.07	0.01	0.08	
AnniCluster 12
Concept	Contribution %	NAGK	GNPDA1	PIP5K1B	FLJ13052	ITPK1	
Phosphotransferases (Alcohol Group Acceptor)	70.25	0.36	0.03	0.5	0.33	0.5	
Phosphotransferases	9.88	0.22	0	0.09	0.13	0.2	
GNPDA1	4.73	0.07	1	0	0	0	
NAGK	4.51	1	0.07	0	0	0	
Aldose-Ketose Isomerases	2.9	0.06	0.7	0	0	0	
Phosphates	2.31	0.08	0.16	0.11	0	0	
Acetylglucosamine	1.76	0.31	0.09	0	0	0	
Enzymes	0.88	0.05	0.08	0	0.07	0	
AnniCluster 13
Concept	Contribution %	CAD	NEDD4	LANCL1	FACL1	SLC27A2	
Ligases	72.22	0.41	0.34	0.4	0.3	0.26	
Coenzyme A Ligases	17.06	0	0	0	0.46	0.59	
Fatty Acids	4.56	0	0	0	0.22	0.34	
Membrane Proteins	2.86	0	0.01	0.26	0	0.15	
Saccharomyces cerevisiae Proteins	1.4	0	0	0	0.05	0.43	
AnniCluster 14
HLA MHC2
Concept	Contribution %	HLA-DQB1	HLA-DRB3	HLA-DRB1	HLA-DPA1	
HLA-DRB1	30.01	0.17	0.26	1	0.07	
Alleles	18.73	0.27	0.22	0.3	0.18	
HLA-DQA1	13.4	0.53	0.11	0.19	0.1	
DRB1	10.43	0.15	0.21	0.4	0.04	
HLA-DQB1	8.45	1	0.05	0.07	0.04	
Antigens	8.07	0.17	0.14	0.19	0.15	
Haplotypes	2.58	0.11	0.11	0.1	0.05	
Polymorphism (Genetics)	1.49	0.06	0.06	0.08	0.08	
HLA-DRB3	1.11	0.01	1	0.01	0	
Genotype	0.87	0.07	0.05	0.07	0.03	
Genetic Predisposition to Disease	0.75	0.1	0.04	0.08	0	
Histocompatibility Antigens	0.7	0.05	0.06	0.07	0.02	
HLA-DPB1	0.6	0.03	0.02	0.02	0.16	
AnniCluster 15
FCGR1A is monocyte specific!
Concept	Contribution %	FCGR1A	FCGRT	FCAR	
Receptors, Fc	90.5	0.23	0.41	0.41	
Immunoglobulin G	2.52	0.04	0.18	0.01	
Monocytes	1.55	0.08	0	0.08	
IgG1	0.82	0.03	0.06	0.02	
Receptors, IgG	0.81	0.06	0.04	0.01	
AnniCluster 16
Concept	Contribution %	FCGR2A	FCGR3A	FCGR2B	
FCGR2A	41.36	1	0.19	0.2	
FCGR3A	28.63	0.07	1	0.23	
FCGR2B	15.91	0.04	0.13	1	
FCGR3B	6.1	0.05	0.28	0.15	
Polymorphism (Genetics)	1.46	0.01	0.13	0.11	
Antigens	1.01	0.07	0.03	0.08	
Receptors, Fc	0.77	0.15	0.05	0	
Receptors, IgG	0.65	0.07	0.1	0	
Lupus Erythematosus, Systemic	0.54	0.01	0.03	0.13	
AnniCluster 17
Concept	Contribution %	MSH5	DP1	PMS1	MSH6	MSH2	
MSH2	25.1	0.07	0.09	0.17	0.51	1	
MLH1	20.57	0.09	0.13	0.61	0.39	0.33	
MSH6	10.77	0.09	0.01	0.14	1	0.17	
Colorectal Neoplasms, Hereditary Nonpolyposis	6.35	0.05	0.05	0.29	0.26	0.22	
Microsatellite Repeats	5.79	0.01	0.26	0.14	0.19	0.18	
DNA Repair	5.57	0.05	0.01	0.28	0.26	0.22	
Base Pair Mismatch	4.57	0.07	0.04	0.19	0.22	0.2	
PMS1	4.51	0.04	0.01	1	0.1	0.04	
DNA	2.69	0.02	0.08	0.14	0.17	0.14	
PMS2	2.43	0.09	0.01	0.2	0.13	0.09	
Colorectal Cancer	1.77	0	0.05	0.15	0.13	0.12	
Proto-Oncogene Proteins	1.36	0	0.04	0.1	0.08	0.18	
Neoplasm Proteins	1.29	0	0.06	0.14	0.08	0.1	
Germ-Line Mutation	1.22	0.02	0.01	0.12	0.13	0.1	
MSH3	0.88	0.04	0.01	0.05	0.19	0.06	
MSH5	0.63	1	0	0.01	0.01	0	
Cancer	0.53	0	0.03	0.09	0.07	0.06	
DP1	0.53	0	1	0	0	0.01	
AnniCluster 18
Concept	Contribution %	STX11	GOLGIN-67	GORASP1	TGOLN2	
GOLGA2	24.66	0	0.72	0.46	0.01	
Membrane Proteins	15.29	0.17	0.13	0.22	0.16	
trans-Golgi Network	3.23	0.07	0	0	0.43	
Membranes	2.78	0.13	0	0.12	0.05	
Organelles	1.41	0	0.22	0.03	0.05	
Protein Transport	1.25	0.11	0	0.04	0.05	
Endosomes	1.02	0	0.1	0	0.13	
GOLGA1	0.83	0	0.24	0.04	0	
Brefeldin A	0.73	0.07	0	0	0.1	
Vesicular Protein Transport	0.57	0.06	0	0.04	0.03	
Carrier Proteins	0.55	0.06	0	0.04	0.03	
GOLGA4	0.54	0	0.19	0.03	0.01	
Intracellular Membranes	0.53	0.11	0	0	0.05	
AnniCluster 19
Concept	Contribution %	GPR35	C5R1	C3AR1	
C5R1	46.79	0.27	1	0.28	
Complement 5a	23.79	0.11	0.92	0.2	
Receptors, Complement	6.16	0.1	0.08	0.41	
Complement 3a	5.9	0	0.15	0.51	
Anaphylatoxin	5.76	0	0.17	0.45	
Anaphylatoxins	5.76	0	0.17	0.45	
GTP-Binding Proteins	2.17	0.32	0.01	0.08	
Complement	0.93	0	0.14	0.09	
Complement Activation	0.67	0	0.12	0.07	
C3AR1	0.59	0	0.01	1	
AnniCluster 20
CD86 specific for monocytes/macrophages. CD83 reportedly not found on the surface but expressed in these cells. CD14 is strongly expressed on monocytes and derived cells, but also weakly on activated granulocytes. 
Concept	Contribution %	CD86	CD83	CD14	ICAM1	ITGAX	ITGB2	ITGAM	ITGAL	
ITGAM	15.89	0.01	0.01	0.04	0.03	0.25	0.19	1	0.23	
ICAM1	8.82	0.05	0.02	0.01	1	0.05	0.12	0.06	0.23	
Antigens	7.71	0.22	0.11	0.2	0.05	0.14	0.13	0.1	0.14	
ITGAL	7.22	0.01	0	0.01	0.04	0.15	0.1	0.09	1	
ITGAX	6.58	0.04	0.05	0.02	0.01	1	0.04	0.08	0.12	
CD86	6.16	1	0.3	0.02	0.01	0.07	0	0.01	0.01	
CD14	4.84	0.03	0.11	1	0.01	0.06	0.01	0.04	0.02	
Adhesions	4.62	0.01	0.01	0.01	0.24	0.06	0.25	0.12	0.23	
Dendritic Cells	4.17	0.19	0.5	0.04	0.01	0.19	0	0.01	0.01	
Intercellular Adhesion Molecule-1	3.87	0.02	0	0.01	0.79	0.02	0.09	0.04	0.14	
ITGB2	3.69	0	0	0	0.02	0.05	1	0.07	0.09	
CD80	2.51	0.53	0.21	0.02	0.02	0.05	0	0	0.01	
Monocytes	2.33	0.05	0.17	0.2	0.02	0.06	0.03	0.06	0.04	
CD83	1.72	0.08	1	0.02	0	0.02	0	0	0	
T-Lymphocytes	1.5	0.21	0.14	0.02	0.03	0.06	0.01	0.02	0.05	
Integrins	1.44	0	0	0	0.03	0.1	0.19	0.07	0.11	
Macrophage-1 Antigen	1.43	0	0	0.01	0.01	0.05	0.11	0.28	0.06	
Cell Adhesion Molecules	1.34	0	0	0.01	0.18	0.04	0.08	0.04	0.14	
Lymphocyte Function-Associated Antigen-1	1.07	0	0	0	0.09	0.02	0.08	0.04	0.24	
Cell Adhesion	0.75	0	0	0	0.07	0.02	0.15	0.05	0.08	
Leukocytes	0.72	0	0	0.01	0.05	0.04	0.1	0.06	0.07	
Membrane Glycoproteins	0.71	0.19	0.12	0.04	0.01	0.02	0	0.01	0.01	
Cytokines	0.69	0.07	0.08	0.05	0.05	0.03	0.01	0.02	0.02	
Neutrophils	0.66	0	0	0.01	0.02	0.02	0.14	0.12	0.04	
TNFRSF5	0.58	0.16	0.13	0.01	0.01	0.04	0	0	0.01	
AnniCluster 21
Concept	Contribution %	LILRA3	TYROBP	TREM1	PTPNS1	LILRB3	LILRB2	LILRB1	LILRB4	LILRA2	
Receptors, Immunologic	28.04	0.22	0.14	0.39	0.28	0.1	0.29	0.3	0.34	0.33	
LILRB1	20.72	0.06	0	0	0.01	0.11	0.44	1	0.24	0.42	
LILRB2	11.28	0	0	0	0	0.12	1	0.2	0.26	0.21	
LILRA2	6.4	0	0	0	0.01	0.14	0.07	0.07	0.21	1	
LILRB4	5.51	0	0	0	0.01	0.06	0.1	0.04	1	0.23	
LILRB3	4.73	0.08	0	0	0	1	0.05	0.02	0.06	0.15	
TYROBP	3.81	0	1	0.21	0.02	0	0.01	0	0	0.05	
Membrane Glycoproteins	1.89	0	0.04	0.28	0.23	0	0	0.04	0.04	0.07	
PTPNS1	1.54	0	0.01	0.02	1	0	0	0.01	0.05	0.05	
Monocytes	1.27	0	0.02	0.09	0.01	0	0.09	0.05	0.15	0.14	
Leukocytes	1.04	0.16	0	0	0	0.07	0.08	0.05	0.07	0.05	
Killer Cells, Natural	0.95	0.08	0.06	0	0	0.04	0.07	0.17	0.04	0	
LILRA3	0.93	1	0	0	0	0.06	0	0.01	0	0	
Immunoglobulins	0.93	0.07	0	0.04	0.03	0	0.07	0.05	0.05	0.12	
Tyrosine	0.91	0	0.16	0.02	0.11	0	0.02	0.02	0.05	0.08	
Antigens	0.79	0	0.01	0	0.04	0	0.06	0.12	0.09	0.11	
Killer Cells	0.63	0.11	0.01	0	0	0	0.01	0.06	0.09	0.1	
LILRA1	0.56	0	0	0	0	0.07	0.03	0.02	0.17	0.08	
AnniCluster 22
Concept	Contribution %	HLA-C	HLA-F	HLA-G	HLA-E	
HLA-A	9.18	0.35	0.2	0.1	0.11	
Histocompatibility Antigens	8.7	0.08	0.08	0.33	0.25	
HLA-C	8.37	1	0.05	0.05	0.07	
HLA-F	5.87	0	1	0.03	0.08	
HLA-B	3.31	0.38	0.07	0.04	0.05	
Antigens	1.85	0.21	0.04	0.05	0.06	
Genes, MHC Class I	1.68	0.03	0.12	0.09	0.08	
Histocompatibility Antigens Class I	1.38	0.04	0.09	0.08	0.08	
Major Histocompatibility Complex	1.16	0.05	0.1	0.04	0.07	
KLRD1	0.67	0.01	0.01	0.04	0.22	
Killer Cells, Natural	0.62	0.04	0.01	0.06	0.09	
KLRC1	0.61	0.01	0.01	0.04	0.2	
Alleles	0.51	0.1	0.04	0.03	0.02	
AnniCluster 23
Concept	Contribution %	TRIF	TLR8	TLR7	TLR5	MYD88	LY96	TLR1	TLR4	TLR2	TOLLIP	IRAK3	
Receptors, Cell Surface	10.22	0.21	0.24	0.28	0.26	0.22	0.24	0.28	0.35	0.36	0.15	0.12	
MYD88	9.2	0.43	0.15	0.12	0.08	1	0.1	0.13	0.1	0.11	0.21	0.32	
TLR1	4.09	0.02	0.17	0.15	0.18	0.06	0.04	1	0.05	0.08	0.12	0.07	
Drosophila Proteins	3.21	0	0.06	0.11	0.13	0.14	0.19	0.22	0.23	0.23	0.13	0.09	
TLR7	3.01	0	0.68	1	0.09	0.02	0	0.06	0.01	0.02	0	0	
Lipopolysaccharides	2.62	0.08	0.03	0.04	0.09	0.11	0.24	0.11	0.27	0.16	0.13	0.14	
TLR9	2.21	0	0.41	0.4	0.17	0.08	0.02	0.12	0.06	0.07	0.02	0	
TLR5	1.9	0	0.18	0.13	1	0.02	0.01	0.1	0.03	0.04	0	0	
TLR3	1.62	0.4	0.12	0.23	0.18	0.06	0	0.09	0.04	0.05	0	0	
Signal Transduction	1.58	0.18	0.03	0.03	0.07	0.13	0.06	0.11	0.07	0.07	0.16	0.19	
IRAK1	1.31	0.02	0.03	0.01	0.03	0.14	0.01	0.08	0.02	0.01	0.3	0.46	
LY96	1.22	0.02	0	0	0.03	0.06	1	0.04	0.11	0.05	0.02	0	
IL1R1	1.17	0.2	0.04	0.04	0.02	0.14	0	0.17	0.02	0.02	0.14	0.16	
Receptors, Interleukin-1	1.16	0.2	0.04	0.04	0.02	0.13	0	0.17	0.02	0.02	0.14	0.16	
NF-kappa B	1.15	0.08	0.07	0.04	0.08	0.11	0.06	0.1	0.07	0.09	0.1	0.11	
NFKB1	0.95	0.08	0.05	0.04	0.07	0.11	0.05	0.09	0.07	0.08	0.08	0.12	
Receptors, Immunologic	0.86	0.17	0.05	0.05	0.02	0.26	0.02	0.05	0.03	0.05	0.07	0.07	
TLR8	0.85	0	1	0.2	0.04	0.01	0	0.02	0	0.01	0	0	
Antigens, Differentiation	0.83	0.15	0.05	0.06	0.02	0.26	0.02	0.04	0.03	0.04	0.07	0.07	
TLR6	0.74	0.03	0.19	0.09	0.09	0.02	0.03	0.18	0.02	0.06	0.06	0	
Cytokines	0.65	0.05	0.08	0.13	0.06	0.07	0.03	0.07	0.08	0.09	0.04	0	
TOLLIP	0.54	0.03	0	0	0	0.02	0	0.02	0.01	0	1	0.09	
AnniCluster 24
phosphotransferases incl protein kinase C / MAPK's
Concept	Contribution %	GNB2L1	HINT1	PRKCQ	PRKCD	PRKCN	NLK	MAPK12	MAPK7	MAPK3	MAP2K1	MAP4K5	MAP7	LTK	SYK	SH3BP2	GRB2	FGR	PIK3R1	IRS2	DOK2	BRDG1	PLEK	NEDD9	FYB	MATK	
Phosphotransferases	15.35	0.14	0.04	0.09	0.14	0.16	0.23	0.17	0.19	0.25	0.29	0.16	0.09	0.06	0.09	0.06	0.09	0.08	0.18	0.08	0.03	0	0.09	0	0.01	0.12	
Mitogen-Activated Protein Kinases	10.78	0	0.01	0.02	0.02	0	0.29	0.39	0.38	0.58	0.4	0.15	0.11	0.02	0.02	0	0.09	0.01	0.01	0.04	0.02	0	0.01	0	0	0	
Signal Transduction	8.65	0.05	0.03	0.05	0.04	0.1	0.07	0.02	0.05	0.12	0.11	0.19	0.01	0.06	0.12	0.08	0.15	0.1	0.06	0.13	0.23	0	0.06	0.1	0.11	0.06	
Tyrosine	8.15	0.01	0	0.01	0.01	0	0	0	0.01	0.03	0.01	0	0	0.09	0.21	0.13	0.23	0.22	0.08	0.15	0.19	0.3	0.04	0.14	0.09	0.15	
Protein-Tyrosine Kinase	6.96	0.01	0.01	0.01	0.01	0	0	0.04	0.01	0.06	0.1	0	0.01	0.25	0.3	0.08	0.12	0.15	0.04	0.04	0.14	0.17	0.04	0	0.07	0.28	
Protein Kinase C	4.46	0.2	0.35	0.35	0.44	0.13	0	0.01	0.01	0.03	0.03	0	0	0.02	0	0	0	0	0	0	0	0	0.02	0	0	0	
PLEK	3.75	0	0	0	0	0.05	0	0	0	0	0	0	0	0	0	0.04	0.01	0.01	0	0.01	0.1	0.54	1	0	0	0	
Phosphoproteins	3.26	0.01	0	0.01	0	0	0	0	0	0.01	0	0	0	0.02	0.01	0.06	0.08	0.05	0.05	0.33	0.28	0	0.14	0.09	0.21	0	
FGR	3.15	0	0	0	0	0	0	0	0	0	0	0	0	0	0.02	0.05	0.04	1	0.03	0.01	0.06	0.14	0.01	0.08	0.06	0.08	
Enzyme Activation	2.21	0.03	0.04	0.04	0.07	0.04	0	0.08	0.07	0.13	0.12	0.17	0.04	0.01	0.03	0.02	0.04	0.02	0.04	0.03	0.05	0	0.04	0	0	0	
GRB2	2.19	0	0	0	0	0	0	0	0	0.01	0.01	0.05	0	0	0.01	0.12	1	0.07	0.03	0.04	0	0	0.01	0.07	0	0	
Signal Pathways	2.1	0.03	0.02	0.02	0.02	0	0.14	0.05	0.05	0.09	0.09	0	0.01	0.03	0.02	0.04	0.08	0.02	0.02	0.06	0.04	0	0.02	0.15	0.01	0.03	
Protein-Serine-Threonine Kinases	1.99	0.01	0	0.02	0.01	0.15	0.11	0.08	0.03	0.07	0.21	0.21	0.02	0.01	0	0	0.02	0	0.02	0.05	0	0	0.05	0	0	0	
1-Phosphatidylinositol 3-Kinase	1.96	0	0	0.01	0.01	0	0	0	0.01	0.05	0.05	0	0	0.02	0.01	0.08	0.08	0.05	0.44	0.24	0	0	0.07	0	0	0	
MAPK3	1.74	0	0.01	0	0	0	0	0.04	0.16	1	0.18	0	0	0.01	0	0	0.02	0	0	0.01	0	0	0	0	0	0	
MAP2K1	1.45	0	0	0	0	0	0.08	0.03	0.09	0.08	1	0	0.06	0	0	0	0.01	0	0	0	0	0	0	0	0	0	
Mitogen-Activated Protein Kinase Kinases	1.24	0	0	0	0	0	0.06	0.1	0.13	0.1	0.31	0	0.17	0	0	0	0.01	0	0	0.01	0	0	0	0	0	0	
MAPK1	1.19	0	0.01	0	0	0	0	0.05	0.15	0.54	0.18	0	0.01	0.01	0	0	0.03	0	0	0.01	0	0	0	0	0	0	
Protein Kinases	1	0.01	0.03	0.01	0.01	0.18	0	0.13	0.04	0.05	0.06	0.13	0.03	0.03	0.01	0	0	0.01	0.01	0	0	0	0.01	0	0	0	
Isoenzymes	0.95	0.06	0.01	0.26	0.26	0.15	0	0.02	0	0	0	0	0	0	0.01	0	0	0	0	0	0	0	0.01	0	0	0	
Proto-Oncogene Proteins	0.69	0	0	0.01	0	0	0.06	0	0.01	0.03	0.03	0.05	0	0.01	0	0.04	0.07	0.06	0.02	0.05	0	0	0.03	0.08	0.05	0	
Phosphotyrosine	0.59	0	0	0	0	0	0	0	0	0.01	0	0	0	0.02	0.05	0.05	0.08	0.14	0.08	0.07	0.04	0	0.02	0	0	0	
MAPK7	0.58	0	0	0	0	0	0.05	0.07	1	0.01	0.01	0	0.01	0	0	0	0	0	0	0	0	0	0	0	0	0	
Receptor Protein-Tyrosine Kinases	0.58	0	0	0	0	0	0	0	0.01	0.01	0.01	0	0	0.08	0.11	0	0.07	0.04	0.03	0.01	0.05	0	0.01	0	0.02	0.11	
Ca(2+)-Calmodulin Dependent Protein Kinase	0.57	0	0.01	0	0.01	0	0	0.14	0.04	0.1	0.09	0.12	0.01	0.01	0	0	0.04	0	0	0.01	0	0	0	0	0	0	
src-Family Kinases	0.56	0.04	0	0.01	0	0	0	0	0.02	0.01	0.01	0	0	0.01	0.07	0.03	0.03	0.07	0.01	0	0	0	0.01	0	0.11	0.1	
FYB	0.52	0	0	0	0	0	0	0	0	0	0	0	0	0	0	0	0	0	0	0	0	0	0	0.12	1	0	
IRS1	0.51	0	0	0.01	0	0	0	0	0	0.01	0	0	0	0.01	0	0	0.05	0.03	0.09	0.54	0	0	0.02	0	0	0	
MAP2K7	0.51	0	0	0	0	0	0.1	0.04	0.05	0	0.13	0.06	0.18	0	0	0	0	0	0	0	0	0	0	0	0	0	
AnniCluster 25
Concept	Contribution %	ARHGAP5	PAK1	TIAM1	ARHGEF3	ARHG	ARHGEF11	
Guanine Nucleotide Exchange Factors	34.38	0.45	0.09	0.29	0.21	0.13	0.38	
RAC1	15.28	0.04	0.35	0.22	0.09	0.27	0.07	
CDC42	13.08	0.04	0.35	0.14	0.09	0.3	0.07	
GTP Phosphohydrolases	8.64	0.19	0.07	0.16	0.07	0.22	0.06	
ARHA	8.52	0.09	0.21	0.08	0.09	0.24	0.07	
ARHG	6.05	0	0	0.01	0.15	1	0	
rho GTP-Binding Proteins	4.16	0.17	0.1	0.05	0	0.16	0.08	
PLEK	2.15	0	0	0.1	0.1	0.03	0.18	
Stress Fibers	1.09	0	0	0.01	0.08	0.05	0.15	
Signal Transduction	0.67	0.08	0.08	0.05	0	0.02	0	
Phosphotransferases	0.54	0	0.27	0.04	0	0.01	0	
Signal Pathways	0.54	0.08	0	0.03	0	0.02	0.08	
ARHGEF3	0.53	0	0	0	1	0.01	0	
AnniCluster 26
Concept	Contribution %	CENTG2	CENTD2	CENTB1	ARF6	ARHGAP8	GRAF	
GTPase-Activating Proteins	22.73	0.5	0.29	0.21	0.09	0.5	0.21	
RASA1	22.68	0.5	0.29	0.21	0.09	0.5	0.21	
ARF1	11.9	0.64	0.17	0.28	0.28	0	0	
ARF6	11.27	0.18	0	0.3	1	0	0	
ADP-Ribosylation Factor 1	5	0.37	0.16	0.12	0.21	0	0	
CENTG2	4.62	1	0.27	0	0.01	0	0	
CENTD2	4.47	0.27	1	0	0	0	0	
MRIP2	2.68	0.24	0.24	0.18	0.02	0	0	
CDC42	1.96	0	0.12	0	0.01	0.29	0.13	
RASA3	1.75	0.19	0.19	0	0.01	0.19	0	
Carrier Proteins	1.61	0.13	0.25	0.08	0.05	0	0	
ARF5	1.53	0.21	0	0.16	0.13	0	0	
Brefeldin A	1.19	0.29	0.12	0	0.07	0	0	
Monomeric GTP-Binding Proteins	0.78	0	0	0	0.07	0.28	0.05	
Stress Fibers	0.53	0.12	0.12	0	0.01	0	0.05	
AnniCluster 27
Concept	Contribution %	WEE1	CCNT2	CCNG1	CCND3	CCND2	CDK6	CDK4	CDKN2D	FLJ20174	CDKN1A	CKS2	GADD45B	GADD45A	
Cyclin-Dependent Kinases	15.48	0.06	0.43	0.29	0.19	0.19	0.48	0.47	0.29	0.16	0.17	0.2	0.03	0.02	
Cell Cycle	11.72	0.2	0.07	0.3	0.25	0.23	0.27	0.28	0.2	0.1	0.21	0.09	0.1	0.13	
CDK4	6.85	0.02	0.04	0.12	0.18	0.18	0.54	1	0.11	0	0.02	0	0	0	
CDKN1A	4.82	0.01	0	0.05	0.01	0.01	0.03	0.03	0.05	0.5	1	0	0.04	0.11	
Cell Cycle Proteins	4.8	0.27	0	0.1	0.13	0.09	0.17	0.15	0.34	0	0.06	0.22	0.03	0.02	
GADD45A	2.63	0.01	0	0	0	0	0	0	0	0.07	0.02	0	0.35	1	
Phosphotransferases	2.56	0.2	0.13	0.16	0.07	0.07	0.17	0.14	0.05	0	0.04	0.11	0.05	0.01	
CDK2	2.48	0.04	0.04	0.16	0.1	0.12	0.26	0.27	0.02	0	0.06	0.12	0	0	
CDC2	2.35	0.32	0	0.11	0.06	0.06	0.11	0.14	0.02	0	0.04	0.23	0.03	0.01	
CCND2	2.13	0	0	0.05	0.17	1	0.08	0.05	0.04	0	0	0.02	0	0	
CDK6	2.11	0.01	0	0.05	0.09	0.09	1	0.16	0.07	0	0.01	0	0	0	
CCND1	1.76	0	0	0.19	0.2	0.15	0.15	0.24	0.03	0	0.04	0	0	0.01	
Cyclin D1	1.74	0	0	0.19	0.2	0.15	0.16	0.24	0.02	0	0.04	0	0	0.01	
CCND3	1.56	0	0	0.03	1	0.16	0.08	0.05	0	0	0	0	0	0	
CCNG1	1.33	0	0	1	0.05	0.07	0.06	0.05	0	0	0.02	0.02	0	0.01	
TP53	1.29	0.02	0	0.04	0.01	0.01	0.02	0.04	0.04	0.17	0.25	0	0.02	0.21	
CCNA2	1.1	0.02	0.14	0.1	0.08	0.07	0.1	0.09	0.01	0.05	0.04	0.09	0	0	
Cyclin A	1.08	0.02	0.14	0.1	0.08	0.07	0.09	0.09	0.01	0.05	0.04	0.09	0	0	
CCNE1	1.01	0.01	0.04	0.2	0.1	0.1	0.14	0.15	0	0	0.04	0	0	0	
Cyclin E	0.98	0.01	0.04	0.2	0.1	0.1	0.14	0.15	0	0	0.03	0	0	0	
Retinoblastoma Protein	0.95	0	0	0.14	0.1	0.07	0.18	0.17	0.04	0	0.04	0	0.01	0	
CDKN2A	0.74	0	0	0.03	0.03	0.03	0.18	0.2	0.22	0	0.02	0	0	0	
CDKN1B	0.74	0.01	0	0.08	0.1	0.08	0.1	0.09	0.13	0	0.05	0	0	0	
Protein p16	0.71	0	0	0.02	0.02	0.02	0.17	0.16	0.31	0	0.02	0	0	0	
Protein-Serine-Threonine Kinases	0.68	0.05	0.09	0.08	0.04	0.04	0.13	0.08	0.03	0	0.02	0.07	0	0	
S Phase	0.63	0.02	0	0.14	0.1	0.07	0.08	0.09	0	0	0.04	0.04	0	0.01	
Tumor Suppressor Proteins	0.61	0	0	0.07	0.08	0.05	0.12	0.09	0.13	0	0.04	0	0	0.01	
Apoptosis	0.5	0.02	0	0.01	0.02	0.02	0.02	0.02	0.04	0	0.1	0	0.14	0.11	
AnniCluster 28
58 nuclear proteins/transcription factors. The table is too large to be pasted in Word but can readily be reproduced using the Anni program. 
AnniCluster 29
17 genes, 
Concept	Contribution %	DATF1	MALT1	BIRC3	PRSS25	BID	BCL3	BCL7A	BCL2A1	MCL1	BCL2	CASP6	CASP3	CASP2	CASP1	NALP1	RIPK2	ANXA5	
Apoptosis	26.47	0.15	0.02	0.22	0.18	0.19	0.1	0.17	0.23	0.26	0.36	0.26	0.35	0.25	0.07	0.15	0.08	0.27	
Caspases	18.33	0.23	0.02	0.18	0.19	0.14	0.08	0.03	0.08	0.09	0.09	0.23	0.45	0.26	0.11	0.38	0.14	0.09	
BCL2	13.6	0	0.01	0.04	0.05	0.14	0.18	0.3	0.23	0.34	1	0	0.08	0.08	0.02	0	0	0.02	
Proto-Oncogene Proteins c-bcl-2	7.94	0	0	0.02	0.03	0.08	0.14	0.3	0.23	0.34	0.55	0	0.07	0.05	0.03	0	0	0.02	
CASP3	3.93	0	0	0	0	0	0	0	0	0.01	0.01	0.24	1	0.28	0.1	0	0	0	
Caspase 1	3.86	0	0	0.01	0	0	0	0	0	0	0.01	0.09	0.24	0.22	0.5	0.25	0.07	0	
Cell Death	3.31	0.2	0	0.05	0.11	0.24	0.03	0	0.06	0.06	0.1	0	0.14	0.14	0	0	0.01	0.06	
CASP2	2.67	0	0	0	0	0	0	0	0	0	0	0.3	0.06	1	0.1	0	0	0	
BIRC3	2.4	0	0.38	1	0.04	0	0	0	0.04	0	0	0	0	0	0	0	0	0	
Cysteine Endopeptidases	1.29	0	0	0.01	0.01	0	0	0	0	0.01	0.01	0.15	0.25	0.22	0.14	0	0.01	0	
BCL2L1	1.22	0	0	0.03	0	0.12	0.04	0	0.19	0.19	0.09	0	0.03	0.04	0	0	0	0.01	
Proto-Oncogene Proteins	1.01	0	0	0.02	0.01	0.06	0.17	0.04	0.07	0.09	0.13	0	0.02	0.03	0	0	0	0.01	
RIPK2	0.94	0	0	0	0	0	0	0	0	0	0	0	0	0	0	0.18	1	0	
FKSG2	0.81	0	0	0.29	0.17	0.14	0	0	0.02	0.01	0.02	0	0	0	0	0	0	0	
MCL1	0.74	0	0	0.01	0.01	0	0	0	0.08	1	0.02	0	0.01	0.01	0	0	0	0	
BIRC2	0.61	0	0.04	0.38	0.03	0	0	0	0.06	0	0	0.12	0	0	0	0	0	0	
Death	0.59	0	0	0.03	0.02	0.11	0.01	0	0.03	0.02	0.04	0	0.06	0.05	0.04	0.05	0.03	0.02	
NF-kappa B	0.57	0	0.05	0.07	0	0	0.08	0	0.08	0.01	0	0	0	0	0	0.06	0.15	0	
MALT1	0.55	0	1	0.11	0.01	0	0	0	0	0	0	0	0	0	0	0	0	0	
BCL3	0.55	0	0	0	0	0	1	0.08	0	0	0.01	0	0	0	0	0	0	0	
NFKB1	0.52	0	0.05	0.06	0	0	0.07	0	0.08	0	0	0	0	0	0	0.07	0.15	0	
AnniCluster 30
Concept	Contribution %	IL1RN	TNF	IL6	IL1B	TRAF5	TRAF4	TNFRSF1B	LTBR	TNFSF12	TNFSF10	TIAF1	
TNF	22.83	0.07	1	0.17	0.16	0.19	0.06	0.23	0.15	0.13	0.23	0.13	
Tumor Necrosis Factor	22.81	0.07	1	0.17	0.16	0.19	0.06	0.23	0.15	0.13	0.23	0.13	
Receptors, Tumor Necrosis Factor	17.19	0.02	0.05	0.01	0	0.36	0.27	0.49	0.37	0.16	0.16	0.09	
Cytokines	3.22	0.14	0.23	0.3	0.19	0	0	0.05	0.03	0.02	0.01	0	
Interleukin-1	3.12	0.31	0.16	0.12	0.43	0.01	0	0.01	0	0	0	0	
Apoptosis	2.79	0	0	0	0	0.02	0.09	0.04	0.02	0.12	0.28	0.25	
IL6	2.53	0.06	0.12	1	0.09	0	0	0.01	0	0	0	0	
TRAF5	2.53	0	0	0	0	1	0.19	0.01	0.02	0.01	0	0	
Interleukin-1beta	2.25	0.17	0.07	0.06	0.8	0	0	0	0	0	0	0	
IL1B	1.86	0.1	0.07	0.04	1	0	0	0	0	0	0	0	
Interleukin-6	1.83	0.06	0.11	0.79	0.09	0	0	0.01	0	0	0	0	
NF-kappa B	1.27	0	0.01	0.01	0.01	0.24	0.11	0.04	0.08	0.04	0.02	0	
TNFSF10	1.23	0	0	0	0	0	0	0	0.01	0.1	1	0	
NFKB1	1.14	0	0.01	0.01	0	0.24	0.12	0.04	0.08	0.04	0.02	0	
TNFRSF1B	1.06	0	0	0	0	0.07	0	1	0.03	0	0	0	
LTBR	1.05	0	0	0	0	0.05	0.02	0.01	1	0.01	0	0	
TNFRSF1A	1.04	0.01	0.02	0	0	0.02	0.06	0.38	0.07	0.02	0.02	0	
TRAF6	0.96	0	0	0	0	0.34	0.25	0.01	0.01	0	0	0	
Carrier Proteins	0.9	0	0.01	0	0	0.03	0	0	0	0.18	0.03	0.25	
TRAF4	0.86	0	0	0	0	0.08	1	0	0	0	0	0	
TRAF1	0.71	0	0	0	0	0.17	0.33	0.01	0.01	0	0	0	
TRAF3	0.55	0	0	0	0	0.23	0.19	0.01	0.02	0	0	0	
Signal Transduction	0.5	0	0	0	0	0.13	0.07	0.04	0.05	0.03	0.03	0	
AnniCluster 31
Concept	Contribution %	CAPZB	CAPZA2	FSCN1	KLHL2	MACF1	FLNA	CORO1A	KIAA0992	ZYX	VASP	TLN1	
Microfilament Proteins	30.69	0.44	0.23	0.29	0.44	0.3	0.33	0.31	0	0.04	0.1	0.04	
Actins	22.94	0.31	0.08	0.19	0.16	0.23	0.11	0.32	0.27	0.15	0.16	0.16	
CORO1A	4.63	0	0	0.03	0.13	0.08	0.11	1	0	0.01	0.01	0.04	
Actin-Binding Proteins	4.6	0	0	0.03	0.13	0.08	0.11	0.99	0	0.01	0.01	0.04	
Cytoskeleton	3.6	0	0	0	0	0.13	0.08	0.15	0.2	0.1	0.06	0.17	
VASP	3.6	0	0	0	0	0	0	0	0.05	0.21	1	0	
Microfilaments	3.31	0	0	0.12	0	0.13	0.07	0.15	0.09	0.09	0.09	0.07	
CAPZA2	2.88	0.26	1	0	0	0	0	0	0	0	0	0	
Cytoskeletal Proteins	2.47	0	0	0	0	0.06	0.01	0.04	0.27	0.12	0.08	0.17	
Actinin	2.45	0	0	0.03	0	0.12	0.1	0.05	0.2	0.08	0.02	0.12	
ZYX	2.03	0	0	0	0	0	0	0	0.06	1	0.09	0	
Focal Adhesions	1.88	0	0	0	0	0.03	0	0.01	0.07	0.27	0.07	0.24	
Phosphoproteins	1.85	0	0	0	0	0.01	0	0.01	0.24	0.1	0.27	0.02	
CAPZB	1.79	1	0.16	0	0	0	0	0	0	0	0	0	
VCL	1.19	0	0	0	0	0	0.01	0.01	0.04	0.15	0.06	0.34	
Vinculin	1.19	0	0	0	0	0	0.01	0.01	0.04	0.15	0.06	0.34	
Carrier Proteins	1.09	0	0	0.15	0.25	0.04	0.01	0.03	0	0.02	0.03	0	
G-Actin	1.02	0.1	0	0.06	0	0.05	0.02	0.1	0	0.04	0.05	0.05	
Stress Fibers	0.98	0	0	0	0.12	0.02	0	0.02	0.18	0.07	0.02	0.06	
Contractile Proteins	0.74	0	0	0	0	0.05	0.3	0.05	0	0.01	0.04	0.01	
AnniCluster 32
Concept	Contribution %	S100A12	S100A9	S100A8	S100A11	S100A6	S100A4	
S100A8	25.23	0.14	0.3	1	0.04	0.02	0.01	
S100A9	21.49	0.2	1	0.12	0.06	0.02	0.01	
eCalcium-Binding Proteins	20.07	0.23	0.16	0.05	0.12	0.25	0.2	
S100A6	12.55	0.05	0.01	0.01	0.08	1	0.09	
S100A4	7.62	0.04	0.01	0	0.03	0.07	1	
S100A12	4.46	1	0.03	0.01	0.02	0.01	0.01	
S100A11	2.86	0.02	0.01	0	1	0.02	0.01	
S100B	1.37	0.09	0	0	0.06	0.08	0.04	
S100A2	0.74	0.06	0.01	0	0.03	0.05	0.06	
S100A1	0.52	0.04	0.01	0	0.02	0.06	0.05	
AnniCluster 33
Concept	Contribution %	CYP27A1	POR	AHR	CYP1B1	ZNF83	PPIF	
Cytochrome P-450	29.29	0.26	0.36	0.07	0.21	0.12	0.27	
CYP1A1	10.59	0	0.02	0.15	0.48	0.16	0.05	
Enzymes	10.24	0.18	0.22	0.03	0.13	0.05	0.17	
Cytochromes	7.48	0.04	0.08	0.07	0.21	0.09	0.16	
Aryl Hydrocarbon Hydroxylases	5.43	0	0.02	0.05	0.36	0.17	0.03	
Receptors, Aryl Hydrocarbon	5.36	0	0	0.95	0.12	0	0.02	
Steroid Hydroxylases	4.94	0.46	0.02	0	0	0.19	0	
AHR	4.47	0	0	1	0.12	0	0	
CYP2A	3.53	0	0	0	0	0.15	0.42	
Hydroxylases	1.96	0.12	0.04	0	0.01	0.17	0.01	
Tetrachlorodibenzodioxin	1.81	0	0	0.44	0.11	0	0	
Cytochrome P-450 CYP1A1	1.58	0	0.01	0.15	0.21	0	0.02	
CYP1B1	1.47	0	0	0.03	1	0	0.01	
PYGL	1.37	0.08	0.07	0.03	0.01	0.05	0.04	
Liver	1.37	0.08	0.07	0.03	0.01	0.05	0.04	
Xenobiotics	0.9	0	0.02	0.07	0.06	0	0.1	
Microsomes, Liver	0.79	0.01	0.27	0.01	0.01	0	0.03	
Metabolism	0.59	0.03	0.03	0.01	0.04	0	0.09	
AnniCluster 34
Concept	Contribution %	RPS2	RPS5	C15orf15	RPS21	RPS7	RPS3A	RPS17	RPL35A	RPL22	RPL37A	RPLP0	
Ribosomal Proteins	82.94	0.2	0.31	0.39	0.45	0.46	0.39	0.47	0.34	0.32	0.4	0.27	
Introns	2.12	0.05	0	0.02	0.17	0.12	0	0.15	0.15	0	0.05	0	
RPS17	2.08	0	0	0	0	0	0	1	0.15	0.05	0	0	
RPS3A	1.78	0	0	0	0	0.16	1	0	0	0	0	0	
RPL22	1.55	0	0	0	0	0	0	0.14	0	1	0	0	
DNA, Complementary	1.48	0	0.1	0.01	0.15	0.1	0.03	0	0.13	0	0.07	0	
RPL35A	1.4	0	0	0	0	0	0	0.15	1	0	0	0	
Pseudogenes	1.23	0	0	0.03	0.11	0.08	0	0.14	0.24	0	0	0	
RPL4	0.57	0	0	0	0	0.19	0.02	0.15	0	0.05	0	0	
Ribosomes	0.55	0.04	0	0.09	0	0	0.02	0	0	0.05	0	0.15	
AnniCluster 35
Concept	Contribution %	SLCO3A1	MVP	ABCC1	ABCC4	
ABCC1	35.65	0.16	0.26	1	0.52	
Multidrug Resistance-Associated Proteins	21.42	0	0.24	0.85	0.45	
P-Glycoproteins	7.87	0	0.28	0.39	0.22	
Drug Resistance, Multiple	5.33	0	0.33	0.3	0.12	
ABCC2	4.47	0.19	0.03	0.15	0.31	
P-Glycoprotein	4.38	0	0.28	0.35	0.07	
ABCC4	4.27	0.11	0.01	0.03	1	
ABCC3	3.61	0.22	0.01	0.06	0.37	
ABCC5	2.03	0.11	0.01	0.04	0.44	
MVP	1.3	0	1	0.03	0.01	
Drug Resistance, Neoplasm	1.21	0	0.18	0.12	0.05	
ABCB1	1.18	0.06	0.09	0.13	0.05	
Vault Ribonucleoprotein Particles	1	0	0.65	0.05	0	
Drug Resistance	0.86	0	0.14	0.09	0.06	
Organic Anion Transporters	0.6	0.31	0	0.02	0.05	
Pharmaceutical Preparations	0.52	0	0.07	0.1	0.06	
AnniCluster 36
Concept	Contribution %	RCP	SFT	TFRC	TFR2	
TFRC	32.45	0.17	0.19	1	0.4	
Receptors, Transferrin	29.86	0.17	0.19	0.88	0.4	
In-Transferrin	7.53	0.23	0.17	0.21	0.16	
Transferrin	6.33	0.23	0.14	0.19	0.14	
TF	6.33	0.23	0.14	0.19	0.14	
Iron	5.79	0	0.34	0.14	0.22	
HFE	3.35	0	0.25	0.02	0.31	
Hemochromatosis	2.33	0	0.21	0.01	0.25	
Iron Overload	1.18	0	0.11	0.01	0.23	
Carrier Proteins	0.99	0.15	0.18	0	0	
Membrane Proteins	0.81	0.15	0.05	0.01	0.06	
Hela Cells	0.78	0.31	0.07	0	0	
AnniCluster 37
Concept	Contribution %	MT1E	MT1F	MT1X	MT2A	
Metallothionein	47.42	0.4	0.38	0.48	0.13	
MT2A	34.95	0.07	0.16	0.18	1	
MT1S	3.57	0.09	0.07	0.17	0.05	
Cadmium	3.25	0.12	0.15	0.05	0.04	
Zinc	2.97	0.16	0.07	0.1	0.02	
Protein Isoforms	2.09	0.09	0.06	0.15	0.01	
MT3	1.38	0	0.07	0.13	0.05	
Metals	0.61	0	0.1	0.04	0.03	
Metals, Heavy	0.57	0.03	0.08	0.04	0.01	
AnniCluster 38
Concept	Contribution %	LRP1	LRPAP1	MRC2	LR8	LDLR	
LRP1	21.24	1	0.36	0.12	0.03	0.03	
LDL-Receptor Related Protein 1	20.42	0.7	0.37	0.11	0.11	0.04	
LDLR	18.46	0.11	0.11	0.06	0.19	1	
Receptors, LDL	17.11	0.11	0.1	0.06	0.19	0.94	
MRC2	3.64	0.04	0.05	1	0	0.01	
Endocytosis	2.14	0.07	0.05	0.22	0.03	0.03	
Receptors, Immunologic	1.95	0.19	0.17	0.05	0	0.01	
Ligands	1.81	0.08	0.07	0.11	0.08	0.02	
Lipoproteins	1.69	0.05	0.05	0.02	0.07	0.2	
LDL-Receptor Related Protein 2	1.52	0.02	0.04	0.18	0.08	0.01	
Heymann Nephritis Antigenic Complex	1.18	0.03	0.12	0.1	0.03	0.01	
LRPAP1	0.96	0.02	1	0.01	0	0	
Lipoproteins, LDL	0.78	0.04	0.01	0.01	0	0.34	
alpha 2-Glucoproteins	0.75	0.13	0.14	0.01	0	0	
Apolipoproteins E	0.74	0.11	0.08	0.01	0	0.06	
alpha-Macroglobulins	0.74	0.12	0.16	0.01	0	0	
LRP2	0.66	0.03	0.17	0.05	0	0.01	
APOE	0.64	0.1	0.08	0.01	0	0.05	
LDL-Receptor Related Protein-Associated Protein	0.64	0.04	0.08	0.04	0.04	0.01	
A2M	0.5	0.1	0.13	0	0	0	
AnniCluster 39
Concept	Contribution %	MMP2	TIMP2	TIMP1	ILF3	
Matrix Metalloproteinases	9.33	0.45	0.4	0.35	0.09	
Tissue-Inhibitor of Metalloproteinase-1	6.71	0.13	0.32	0.72	0.05	
Tissue Inhibitor-of Metalloproteinase-2	6.68	0.18	0.84	0.24	0.04	
Tissue Inhibitor of Metalloproteinases	4.96	0.14	0.34	0.38	0.09	
MMP9	4.59	0.39	0.23	0.24	0.06	
Metalloendopeptidases	4.24	0.27	0.3	0.22	0.08	
Gelatinase B	3.95	0.35	0.21	0.23	0.06	
Gelatinases	2.19	0.26	0.18	0.11	0.07	
MMP1	2.17	0.16	0.18	0.22	0.06	
Interstitial Collagenase	1.77	0.13	0.15	0.2	0.06	
Collagenases	1.29	0.12	0.11	0.14	0.08	
MMP3	0.89	0.09	0.09	0.13	0.07	
Stromelysin 1	0.85	0.08	0.09	0.13	0.06	
Extracellular Matrix	0.63	0.11	0.11	0.1	0.02	
AnniCluster 40
Concept	Contribution %	UPP1	DPYD	TYMS	HSRTSBETA	
DPYD	2.99	0.04	1	0.03	0	
Floxuridine	2.65	0.11	0.02	0.06	0.33	
Enzymes	1.09	0.08	0.08	0.04	0.07	
Thymidine Phosphorylase	1.07	0.23	0.09	0.02	0	
Uracil	0.84	0.16	0.12	0.01	0	
UPP1	0.66	1	0.01	0.01	0	
Uridine Phosphorylase	0.66	1	0.01	0.01	0	
Pyrimidines	0.53	0.14	0.07	0.02	0	
AnniCluster 41
Concept	Contribution %	ADORA2B	P2RX7	GPR86	
P2RY5	43.61	0.44	0.54	0.34	
Receptors, Purinergic	40.34	0.44	0.54	0.3	
Adenosine	3.51	0.37	0.05	0.07	
P2RY12	2.85	0	0.09	0.42	
P2RX7	2.27	0	1	0.03	
P2RY1	1.52	0	0.07	0.27	
P2RX1	1.39	0	0.16	0.11	
Adenosine Triphosphate	1.26	0.01	0.26	0.05	
P2RY2	0.62	0	0.08	0.09	
AnniCluster 42
Concept	Contribution %	CTSD	CTSZ	CSTB	CSTA	CST3	CTSS	CTSL	CTSB	CTSH	
Cathepsins	19.67	0.14	0.34	0.04	0.08	0.03	0.49	0.51	0.28	0.42	
CTSB	13.16	0.06	0	0.06	0.16	0.07	0.15	0.27	1	0.4	
Cathepsin B	12.98	0.06	0	0.06	0.17	0.07	0.15	0.27	0.97	0.4	
Cystatins	12.6	0	0	0.41	0.6	0.66	0.07	0.07	0.07	0.12	
Cysteine Endopeptidases	8.62	0.01	0.19	0.06	0.15	0.07	0.24	0.27	0.18	0.38	
CTSL	7.25	0.01	0.04	0.04	0.07	0.02	0.16	1	0.12	0.22	
Cysteine Proteinase Inhibitors	4.55	0	0	0.2	0.34	0.2	0.09	0.11	0.09	0.12	
Endopeptidases	3.25	0.09	0.09	0.01	0.05	0.02	0.14	0.26	0.18	0.13	
CST3	3.06	0	0	0.05	0.14	1	0.06	0.02	0.03	0.03	
CSTA	2.81	0	0	0.15	1	0.05	0.02	0.02	0.03	0.05	
CTSH	2.2	0.01	0	0.03	0.04	0.01	0.05	0.07	0.05	1	
CTSS	2.01	0	0.05	0.01	0.01	0.02	1	0.04	0.02	0.04	
CTSD	1.49	1	0	0	0.01	0	0.02	0.04	0.07	0.05	
Cathepsin D	1.48	0.99	0	0	0.01	0	0.02	0.04	0.07	0.05	
Cystatin A	1.14	0	0	0.11	0.63	0.04	0.01	0.01	0.01	0.02	
Papain	1.1	0	0.07	0.02	0.11	0.04	0.07	0.08	0.05	0.1	
CSTB	1.02	0	0	1	0.07	0.01	0.01	0.01	0	0.02	
Lysosomes	0.5	0.11	0	0	0.01	0	0.03	0.07	0.1	0.09	
