# Supplementary material for: Text-derived concept profiles support assessment of DNA microarray data for acute myeloid leukemia and for androgen receptor stimulation
Source: BMC Bioinformatics. 2007 Jan 18;8:14. doi: 10.1186/1471-2105-8-14 (PMC1784107; doi:10.1186/1471-2105-8-14)
Supplement: Additional File 3 — Annotation of the second DNA microarray dataset. [file 1471-2105-8-14-S3.rtf]

Appendix 3, 
Anni annotation of differentially expressed genes following agonistic stimulation of the androgen receptor.

Genes in Analysis: 129.
In the annotations concepts are shown if their percentage contribution to the average cosine score for the group exceeds 0.5%. 

Annicluster 1 
Concept	Contribution %	RAB27B	MYRIP	MLPH	RAB27A	
RAB27A	52.17	0.61	0.74	0.73	1	
MLPH	11.16	0	0.44	1	0.29	
Myosin Type V	7.22	0.04	0.68	0.4	0.22	
Melanosomes	6.7	0.12	0.3	0.47	0.27	
RAB27B	4.06	1	0.14	0	0.11	
MYRIP	2.98	0.07	1	0.09	0.06	
Melanocytes	2.73	0.13	0.14	0.28	0.17	
Myosins	2.33	0.04	0.38	0.22	0.12	
Myosin Heavy Chains	1.72	0	0.46	0.18	0.09	
GTP Phosphohydrolases	1.31	0.17	0.23	0.04	0.08	
Actins	1.17	0.05	0.32	0.12	0.06	
Exocytosis	0.87	0.08	0.12	0.08	0.12	
Secretory Vesicles	0.68	0.07	0.16	0.06	0.09	
Carrier Proteins	0.59	0	0.11	0.17	0.09	
Organelles	0.54	0.11	0	0.12	0.09	
rab GTP-Binding Proteins	0.52	0.16	0	0.04	0.12	
Annicluster 2
Concept	Contribution %	PRKCM	CAMKK2	SGK	STK39	SNRK	PAK2	CCNG2	CCNH	CCNE2	CaMKIINalpha	PIK3R1	PIK3CB	PIK3R3	
Protein-Serine-Threonine Kinases	23.24	0.07	0.23	0.35	0.22	0.34	0.23	0.04	0.21	0.08	0.04	0.02	0.03	0	
PIK3R1	7.55	0	0	0	0	0	0	0	0	0	0	1	0.24	0.19	
1-Phosphatidylinositol 3-Kinase	6.69	0	0	0.02	0	0	0.01	0	0	0	0	0.44	0.43	0.22	
Protein Kinases	4.94	0.05	0.11	0.03	0.1	0.18	0.08	0.07	0.03	0	0.16	0.01	0.02	0	
Cyclins	2.39	0	0	0	0	0	0	0.19	0.27	0.24	0	0	0	0	
Cyclin-Dependent Kinases	2.23	0	0	0	0	0	0	0.14	0.41	0.17	0	0	0	0	
Cell Cycle	2.11	0	0	0	0	0	0	0.28	0.2	0.18	0	0	0	0	
DNA, Complementary	2.06	0	0.04	0	0.18	0.29	0	0.01	0.01	0.05	0	0	0	0.06	
Ca(2+)-Calmodulin Dependent Protein Kinase	1.97	0	0.36	0	0	0	0.03	0	0	0	0.28	0	0	0	
TLK2	1.45	0.02	0	0.04	0.08	0.47	0.03	0	0	0	0	0	0	0	
CDC2	1.19	0	0	0	0	0	0	0.19	0.17	0.13	0	0	0	0	
Phosphatidylinositols	1.07	0.01	0	0.01	0	0	0	0	0	0	0	0.15	0.23	0.08	
CDK2	1.03	0	0	0	0	0	0	0.05	0.21	0.23	0	0	0	0	
CCNE1	0.98	0	0	0	0	0	0	0.02	0.07	0.42	0	0	0	0.05	
Cyclin E	0.83	0	0	0	0	0	0	0.02	0.07	0.37	0	0	0	0.05	
Enzyme Activation	0.73	0.09	0.08	0	0	0	0.1	0	0.03	0	0	0.04	0	0	
CCNA2	0.65	0	0	0	0	0	0	0.09	0.13	0.15	0	0	0	0	
Signal Transduction	0.64	0.06	0.06	0.01	0	0	0.04	0	0	0	0	0.06	0.08	0	
PI3	0.61	0	0	0	0	0	0	0	0	0	0	0.08	0.15	0.12	
PIK3CB	0.54	0	0	0	0	0	0	0	0	0	0	0.04	1	0	
Cyclin A	0.54	0	0	0	0	0	0	0.08	0.13	0.13	0	0	0	0	
Annicluster 3
Concept	Contribution %	SOCS2	MLL3	MLLT2	ELL2	ELF3	NR5A2	TIF1	NCOA2	GATA2	LEF1	SIM2	MYB	MYC	
DNA	7.88	0.07	0.08	0.06	0	0.09	0.11	0.05	0.01	0.15	0.11	0.05	0.05	0.04	
Trans-Activators	3.88	0.21	0	0	0	0.07	0.03	0.02	0.05	0.04	0.17	0	0.05	0.01	
MYC	3.37	0	0	0	0	0	0	0	0	0	0	0	0.13	1	
MLLT2	3.36	0	0.03	1	0.14	0	0	0	0	0	0	0	0	0	
Transcription, Genetic	3.33	0.05	0.02	0	0	0.03	0.09	0.1	0.06	0.06	0.06	0	0.05	0.03	
Receptors, Nuclear	2.44	0	0.03	0	0	0	0.22	0.13	0.15	0	0	0	0	0	
Proto-Oncogenes	2.3	0	0.11	0.13	0.06	0	0	0	0	0	0	0	0.12	0.08	
Proto-Oncogene Proteins	2.3	0.03	0	0	0	0.23	0	0.01	0	0.02	0.07	0	0.13	0.04	
Translocation (Genetics)	2.12	0	0.12	0.21	0.16	0.01	0	0	0.01	0	0	0	0	0.01	
NCOA2	2.04	0	0	0	0	0	0	0.08	1	0	0	0	0	0	
Promoter (Genetics)	1.83	0.01	0	0	0	0.13	0.1	0.02	0.01	0.08	0.04	0	0.03	0.02	
Nuclear Protein	1.77	0	0	0.07	0	0	0.02	0.12	0.06	0.02	0.02	0.09	0.01	0	
Nuclear Proteins	1.77	0	0	0.07	0	0	0.02	0.12	0.06	0.02	0.02	0.09	0.01	0	
TIF1	1.49	0	0	0	0	0	0	1	0.06	0	0	0	0	0	
Repressor Proteins	1.21	0.21	0	0	0	0	0.04	0.09	0.02	0.01	0.01	0.02	0	0	
Trans-Activation (Genetics)	1.16	0.01	0	0.02	0	0.04	0.03	0.04	0.09	0.03	0.04	0	0.03	0	
Leukemia	1.03	0	0.04	0.17	0.12	0	0	0	0	0.01	0	0	0.03	0	
Transfection	1.01	0.02	0	0	0.08	0.06	0.04	0.02	0.02	0.03	0.02	0	0.02	0.01	
Promoter Regions (Genetics)	0.96	0	0	0	0	0.08	0.07	0.01	0	0.08	0.04	0	0.02	0.01	
MYB	0.87	0	0	0	0	0	0	0	0	0.02	0	0	1	0.02	
Chromosomes, Human, Pair 11	0.79	0	0.19	0.17	0	0.01	0	0	0	0	0	0	0	0	
Oncogene Proteins, Fusion	0.69	0	0.03	0.23	0.09	0	0	0	0	0	0	0	0	0	
Cell Line	0.68	0.03	0	0.01	0.04	0.03	0.01	0.01	0.01	0.02	0.02	0	0.04	0.04	
Gene Expression	0.55	0.03	0	0	0	0.04	0.02	0.01	0.01	0.06	0.02	0	0.02	0.03	
