# Supplementary material for: Text-derived concept profiles support assessment of DNA microarray data for acute myeloid leukemia and for androgen receptor stimulation
Source: BMC Bioinformatics. 2007 Jan 18;8:14. doi: 10.1186/1471-2105-8-14 (PMC1784107; doi:10.1186/1471-2105-8-14)
Supplement: Additional File 4 — Semantic types used for filtering. [file 1471-2105-8-14-S4.rtf]

Appendix 4, 
Semantic types used in concept profiles.
Semantic Type ID	Name	
T017	Anatomical Structure	
T018	Embryonic Structure	
T019	Congenital Abnormality	
T020	Acquired Abnormality	
T021	Fully Formed Anatomical Structure	
T022	Body System	
T023	Body Part, Organ, or Organ Component	
T024	Tissue	
T025	Cell	
T026	Cell Component	
T028	Gene or Genome	
T029	Body Location or Region	
T030	Body Space or Junction	
T031	Body Substance	
T032	Organism Attribute	
T033	Finding	
T034	Laboratory or Test Result	
T037	Injury or Poisoning	
T038	Biologic Function	
T039	Physiologic Function	
T040	Organism Function	
T041	Mental Process	
T042	Organ or Tissue Function	
T043	Cell Function	
T044	Molecular Function	
T045	Genetic Function	
T046	Pathologic Function	
T047	Disease or Syndrome	
T048	Mental or Behavioral Dysfunction	
T049	Cell or Molecular Dysfunction	
T050	Experimental Model of Disease	
T103	Chemical	
T104	Chemical Viewed Structurally	
T109	Organic Chemical	
T110	Steroid	
T111	Eicosanoid	
T114	Nucleic Acid, Nucleoside, or Nucleotide	
T115	Organophosphorus Compound	
T116	Amino Acid, Peptide, or Protein	
T118	Carbohydrate	
T119	Lipid	
T120	Chemical Viewed Functionally	
T121	Pharmacologic Substance	
T122	Biomedical or Dental Material	
T123	Biologically Active Substance	
T124	Neuroreactive Substance or Biogenic Amine	
T125	Hormone	
T126	Enzyme	
T127	Vitamin	
T129	Immunologic Factor	
T130	Indicator, Reagent, or Diagnostic Aid	
T131	Hazardous or Poisonous Substance	
T167	Substance	
T168	Food	
T184	Sign or Symptom	
T190	Anatomical Abnormality	
T191	Neoplastic Process	
T192	Receptor	
T194	Archaeon	
T195	Antibiotic	
T196	Element, Ion, or Isotope	
T197	Inorganic Chemical	
T200	Clinical Drug	
T201	Clinical Attribute	


Excluded semantic types
Semantic Type ID	Name	
T001	Organism	
T002	Plant	
T003	Alga	
T004	Fungus	
T005	Virus	
T006	Rickettsia or Chlamydia	
T007	Bacterium	
T008	Animal	
T009	Invertebrate	
T010	Vertebrate	
T011	Amphibian	
T012	Bird	
T013	Fish	
T014	Reptile	
T015	Mammal	
T016	Human	
T051	Event	
T052	Activity	
T053	Behavior	
T054	Social Behavior	
T055	Individual Behavior	
T056	Daily or Recreational Activity	
T057	Occupational Activity	
T058	Health Care Activity	
T059	Laboratory Procedure	
T060	Diagnostic Procedure	
T061	Therapeutic or Preventive Procedure	
T062	Research Activity	
T063	Molecular Biology Research Technique	
T064	Governmental or Regulatory Activity	
T065	Educational Activity	
T066	Machine Activity	
T067	Phenomenon or Process	
T068	Human-caused Phenomenon or Process	
T069	Environmental Effect of Humans	
T070	Natural Phenomenon or Process	
T071	Entity	
T072	Physical Object	
T073	Manufactured Object	
T074	Medical Device	
T075	Research Device	
T077	Conceptual Entity	
T078	Idea or Concept	
T079	Temporal Concept	
T080	Qualitative Concept	
T081	Quantitative Concept	
T082	Spatial Concept	
T083	Geographic Area	
T085	Molecular Sequence	
T086	Nucleotide Sequence	
T087	Amino Acid Sequence	
T088	Carbohydrate Sequence	
T089	Regulation or Law	
T090	Occupation or Discipline	
T091	Biomedical Occupation or Discipline	
T092	Organization	
T093	Health Care Related Organization	
T094	Professional Society	
T095	Self-help or Relief Organization	
T096	Group	
T097	Professional or Occupational Group	
T098	Population Group	
T099	Family Group	
T100	Age Group	
T101	Patient or Disabled Group	
T102	Group Attribute	
T169	Functional Concept	
T170	Intellectual Product	
T171	Language	
T185	Classification	
T203	Drug Delivery Device	
